# Hippocampal subfield volumetry from structural isotropic 1 mm^3^ MRI scans: A note of caution

**DOI:** 10.1002/hbm.25234

**Published:** 2020-10-15

**Authors:** Laura E. M. Wisse, Gaël Chételat, Ana M. Daugherty, Robin de Flores, Renaud la Joie, Susanne G. Mueller, Craig E. L. Stark, Lei Wang, Paul A. Yushkevich, David Berron, Naftali Raz, Arnold Bakker, Rosanna K. Olsen, Valerie A. Carr

**Affiliations:** ^1^ Diagnostic Radiology Lund University Lund Sweden; ^2^ Penn Image Computing and Science Laboratory, Department of Radiology University of Pennsylvania Philadelphia Pennsylvania USA; ^3^ Penn Memory Center, Department of Neurology University of Pennsylvania Philadelphia Pennsylvania USA; ^4^ Université Normandie, Inserm Université de Caen‐Normandie, Inserm UMR‐S U1237 Caen France; ^5^ Department of Psychology Wayne State University Detroit Michigan USA; ^6^ Institute of Gerontology Wayne State University Detroit Michigan USA; ^7^ Department of Psychiatry and Behavioral Neurosciences Wayne State University Detroit Michigan USA; ^8^ Memory and Aging Center University of California San Francisco San Francisco California USA; ^9^ Department of Radiology University of California San Francisco San Francisco California USA; ^10^ Center for Imaging of Neurodegenerative Diseases San Francisco VA Medical Center San Francisco California USA; ^11^ Department of Neurobiology and Behavior University of California Irvine Irvine California USA; ^12^ Department of Psychiatry and Behavioral Sciences Northwestern University Feinberg School of Medicine Chicago Illinois USA; ^13^ Department of Radiology Northwestern University Feinberg School of Medicine Chicago Illinois USA; ^14^ Clinical Memory Research Unit, Department of Clinical Sciences Malmö Lund University Lund Sweden; ^15^ Center for Lifespan Psychology Max Planck Institute for Human Development Berlin Germany; ^16^ Department of Psychiatry and Behavioral Sciences Johns Hopkins University School of Medicine Baltimore Maryland USA; ^17^ Rotman Research Institute Baycrest Health Sciences Toronto Canada; ^18^ Department of Psychology San Jose State University San Jose California USA

**Keywords:** 1 mm^3^, FreeSurfer, hippocampal subfields, MRI, volumetry

## Abstract

Spurred by availability of automatic segmentation software, in vivo MRI investigations of human hippocampal subfield volumes have proliferated in the recent years. However, a majority of these studies apply automatic segmentation to MRI scans with approximately 1 × 1 × 1 mm^3^ resolution, a resolution at which the internal structure of the hippocampus can rarely be visualized. Many of these studies have reported contradictory and often neurobiologically surprising results pertaining to the involvement of hippocampal subfields in normal brain function, aging, and disease. In this commentary, we first outline our concerns regarding the utility and validity of subfield segmentation on 1 × 1 × 1 mm^3^ MRI for volumetric studies, regardless of how images are segmented (i.e., manually or automatically). This image resolution is generally insufficient for visualizing the internal structure of the hippocampus, particularly the stratum radiatum lacunosum moleculare, which is crucial for valid and reliable subfield segmentation. Second, we discuss the fact that automatic methods that are employed most frequently to obtain hippocampal subfield volumes from 1 × 1 × 1 mm^3^ MRI have not been validated against manual segmentation on such images. For these reasons, we caution against using volumetric measurements of hippocampal subfields obtained from 1 × 1 × 1 mm^3^ images.

## Graphical abstract

In this commentary, we outline our concerns regarding the utility and validity of subfield segmentation on 1 × 1 × 1 mm^3^ MRI for volumetric studies. This image resolution is generally insufficient for visualizing the internal structure of the hippocampus, particularly the stratum radiatum lacunosum moleculare, which is crucial for valid and reliable subfield segmentation. Second, automatic methods that are employed most frequently to obtain hippocampal subfield volumes from 1 × 1 × 1 mm^3^ MRI have not been validated against manual segmentation on such images.

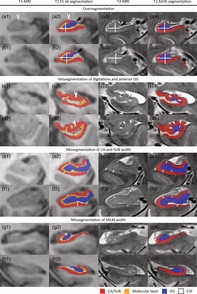

## 1 INTRODUCTION

**FIGURE 1:**
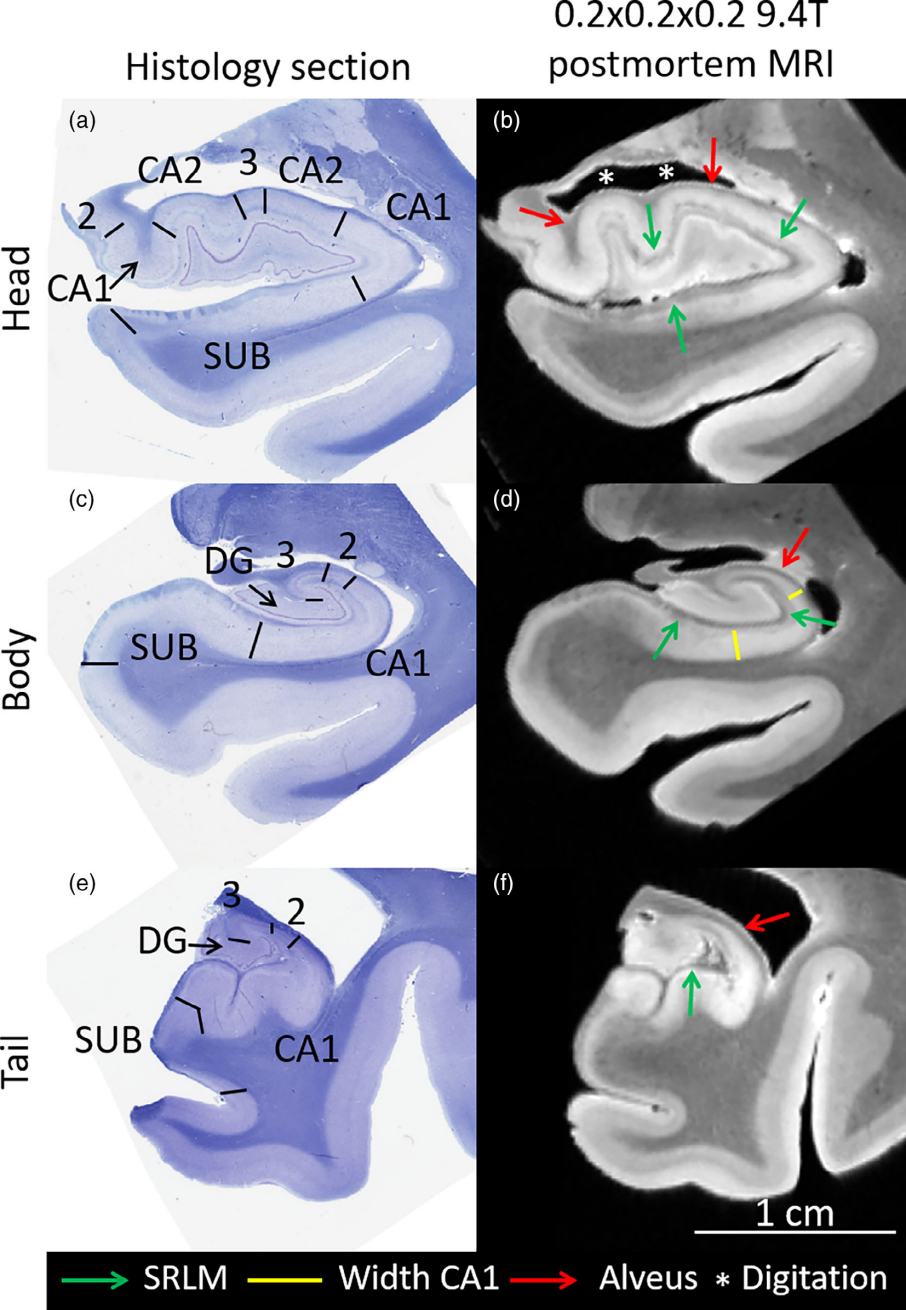
Coronal histology sections (Kluver Barrera stained) and ex vivo 0.2 × 0.2 × 0.2 mm^3^ MRI in the same subject of the hippocampal head (a,b), body (c,d), and tail (e,g). The black lines in the histology sections demarcate the cytoarchitectonic border between the subfields, traced by an expert analyzing digital histology sections at full 0.5 × 0.5 μm^2^ resolution. The green arrows point to the stratum radiatum lacunosum moleculare (SRLM) layer, which appears hypointense in the MRI. Note that the width of the cornu ammonis and subiculum are determined by the location of the SLRM, a critical landmark for segmentation of the subfields (yellow lines). Moreover, on the ex vivo MRI, the alveus (outer hypointense band, red arrows) can be seen, which is helpful for demarcating the outer border of the hippocampus and specifically its digitations (white asterisks). CA, cornu ammonis, SUB, subiculum (includes pre‐ and parasubiculum), DG, dentate gyrus

The subfields of the human hippocampus (as demarcated in Figure 1) have distinct cytoarchitecture, neurochemistry, and function, and each plays a distinct role in multiple cognitive processes, including episodic memory and spatial navigation (Bakker, Kirwan, Miller, & Stark, 2008; Brown, Hasselmo, & Stern, 2014; Carr et al., 2017; Daugherty, Bender, Raz, & Ofen, 2016; Duncan, Tompary, & Davachi, 2014; Kyle, Smuda, Hassan, & Ekstrom, 2015; Yassa & Stark, 2011). Moreover, hippocampal subfields are thought to be selectively vulnerable to several diseases and pathological conditions, such as hypoxia/ischemia, Alzheimer's disease (AD), temporal lobe epilepsy and depression (Braak & Braak, 1991; Goubran et al., 2016; Schmidt‐Kastner & Freund, 1991; Small, Schobel, Buxton, Witter, & Barnes, 2011). Thus, valid and reliable volumetry of hippocampal subfields estimated from MRI may yield useful biomarkers for studying disease mechanisms or for early diagnosis and clinical trials. The interest in in vivo hippocampal subfields research grew out of extensive investigations in animal models and postmortem human studies, which proliferated with the advent of freely available software for automating hippocampal subfield segmentation. In most respects, the development of automatic hippocampal subfield segmentation is a worthy development that strengthens the translational connection between animal and human studies, and between ex vivo and in vivo research. It broadens the community contributing to the study of cognitive and clinical neuroscience and allows for the enhancement of the knowledge base by enabling re‐analysis of archival data. These positive developments, however, come with limitations that need to be critically examined.

**TABLE 1:** Differences in hippocampal subfield volumes in older adults, patients with MCI and patients with AD using automatic FreeSurfer 6.0 segmentation of T_1_‐weighted MRI and manual segmentation of proton density (PD)‐weighted MRI. Bold font indicates significance after Bonferroni correction (0.05/24)

	Older adults vs. MCI		Older adults vs. AD		MCI vs. AD	
FS6‐T1	Effect size (95% CI)	*p*‐value	Effect size (95% CI)	*p*‐value	Effect size (95% CI)	*p*‐value
CA1	1.19 (0.28; 2.10)	.02	2.35 (1.49; 3.21)	**0.000000001**	0.97 (0.07; 2.00)	.01
CA2/3/4/DG^a^	1.59 (0.65; 2.53)	**.001**	2.08 (1.16; 2.91)	**0.00000007**	0.45 (0.54; 1.45)	.25
SUB^b^	1.11 (0.21; 2.01)	.03	2.78 (1.86; 3.70)	**0.00000000003**	1.56 (0.44; 2.67)	**0.0006**
Whole hippocampus	1.56 (0.62; 2.50)	.002	2.70 (1.79; 3.61)^c^	**0.00000000004**	0.93 (0.10; 1.96)	.01
**Man‐PD**						
CA1	1.47 (0.54; 2.40)	**0.0001**	1.62 (0.83; 2.39)	**0.00000008**	0.04 (0.94; 1.03)	.69
CA2/3/4/DG	0.19 (−0.67; 1.05)	.93	1.03 (0.32; 1.75)	0.023	0.65 (0.36; 1.65)	.21
SUB	1.00 (0.11; 1.89)	.06	1.81 (1.02; 2.60)	**0.000002**	0.63 (0.38; 1.64)	.09
Whole hippocampus	1.08 (0.18; 1.98)	.02	1.98 (1.17; 2.80)	**0.0000003**	0.45 (0.46; 1.54)	.11

Abbreviations: AD, Alzheimer's disease; CA, cornu ammonis; CI, confidence interval; DG, dentate gyrus; Man, manual; MCI, Mild Cognitive Impairment; PD, proton density; SUB, subiculum.

^a^ This label includes CA2, CA3, CA4 and GC‐DG.

^b^ This label includes subiculum, presubiculum and parasubiculum.

^c^The slightly higher reported effect sizes are likely due to a previously reported bias in FreeSurfer 6.0 where larger hippocampal volumes are over‐segmented to a larger extent than smaller hippocampal volumes (Schmidt et al., 2018), which could potentially explain the larger effect size in the current study.

Although all available automatic segmentation methods were developed using high resolution images, over 200 peer‐reviewed publications (see Appendix 1) have applied these automatic methods to ~1 × 1 × 1 mm^3^ T1‐weighted images (the actual resolution of these images varies but is close to 1 mm^3^ isotropic; for simplicity, we will refer to this resolution as ~1 mm^3^ isotropic hereafter). Most of these studies investigated hippocampal subfield volumes, and of these, many reported biologically implausible results. For instance, reports of smaller volumes of the cornu ammonis (CA) 4, dentate gyrus (DG), or the granular cell layer of DG in early AD patients compared to controls (Broadhouse et al., 2019; Mak et al., 2017; Marizzoni et al., 2018; Zhao et al., 2019) are at odds with the classic pathological findings showing that these subfields do not accumulate AD pathology (neurofibrillary tangles) until later stages of the disease (Braak & Braak, 1991). Moreover, many of the hippocampal subfield volumetric findings reported in ~1 mm^3^ isotropic MRI studies have not been replicated. For example, a recent review of findings on hippocampal subfield volumes in schizophrenia and bipolar disorder (Haukvik, Tamnes, Söderman, & Agartz, 2018), reported discrepant results in most studies included in the review.

In this commentary, we therefore express our concerns regarding hippocampal subfield volumetry estimated from ~1 mm^3^ isotropic MRI. We note that these concerns are not limited to T_1_‐weighted MRI but rather apply to all sequences with a similar resolution. First, we point out that ~1 mm^3^ isotropic resolution is insufficient for visualizing inner structures of the hippocampus, regardless of how the images are subsequently segmented (i.e., manually or automatically). Second, we review the validation of automatic hippocampal subfield segmentation on ~1 mm^3^ isotropic MRI and, in most instances, the lack thereof. Finally, we express our concerns about alternative, indirect validation approaches of automatic hippocampal subfield segmentation on ~1 mm^3^ isotropic MRI.

## 2 IMAGE RESOLUTION AND VISUALIZATION OF INNER STRUCTURES OF THE HIPPOCAMPUS

**FIGURE 2:**
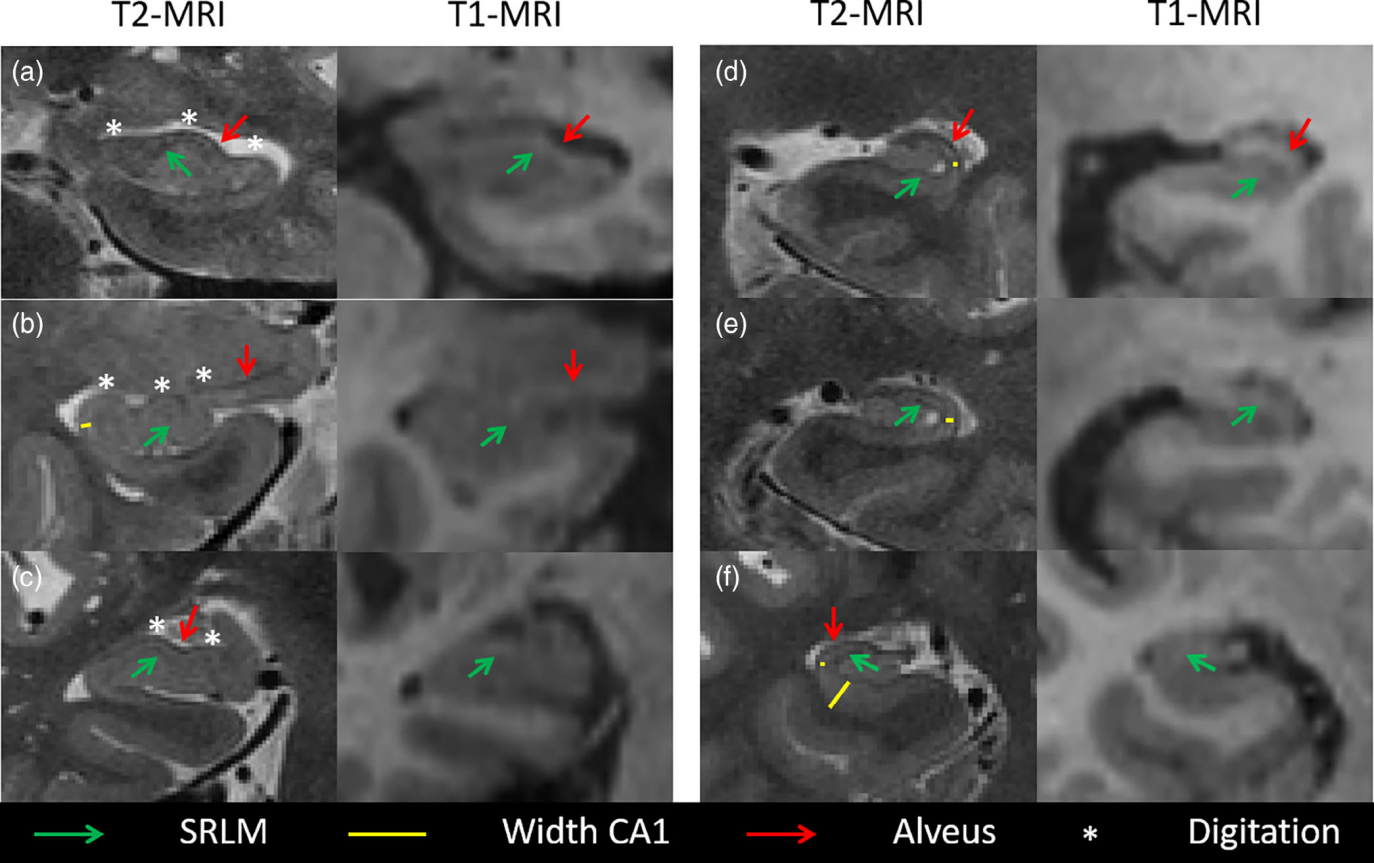
Comparison of 0.4 × 0.4 × 2.6 mm^3^ T_2_‐ and 1 × 1 × 1 mm^3^ T_1_‐weighted MRI in the same subjects demonstrating better visualization of the inner structure of the hippocampus on 0.4 × 0.4 × 2.6 mm^3^ T_2_‐weighted MRI; a and d‐f are cognitively normal (CN) older adults and b‐c are patients with Mild Cognitive Impairment (MCI). The green arrows point out the hypointense band stratum radiatum lacunosum moleculare (SRLM) which can be observed on the high‐resolution T_2_‐weighted MRI scans but not on the 1 mm^3^ isotropic T_1_‐weighted MRI scans. The visualization of SRLM allows for accurate depiction of the width of cornu ammonis (CA) as indicated by the yellow lines in T_2_‐weighted MRI but not on T_1_‐weighted MRI. Similarly, the high resolution and the depiction of SRLM and alveus (outer hypointense band, red arrows) in the T_2_‐weighted MRI, but not T_1_‐weighted MRI, allow for clear visualization of the digitations (white asterisks) in the hippocampal head, which is crucial for accurate segmentation. Note that the alveus is visible as a hyperintense band on T_1_‐weighted MRI but is less sharp than on the T_2_‐weighted MRI

A very important consideration for choosing an imaging protocol is how well the resulting image allows for visualization of the inner structure of the hippocampus, which is critically important for valid and reliable segmentation. Although not all subfield boundaries are apparent on in vivo MRI, one important landmark, the stratum radiatum lacunosum moleculare (SRLM), can be visualized with appropriate tissue contrast and image resolution. The SRLM is a layer of CA and subiculum (Note that the subicular portion of the SRLM lacks the stratum radiatum) (Figures 1 and 2) that spans the entire extent of the hippocampus and is critical for determining a large portion of the borders between these subfields and the DG (Duvernoy et al., 2005; Insausti & Amaral, 2012). It appears as a thin hypointense layer in T_2_‐weighted (or, for example, proton density weighted) MRI, and to a lesser extent, as a hyperintense layer on T_1_‐weighted MRI. Because the SRLM denotes the boundary between the DG and the subiculum and CA regions, visualizing this structure enables the characterization of selective regional atrophy or thinning in these subfields (Figures 1 and 2). In the hippocampal head, identifying SRLM is necessary for visualizing the hippocampal digitations, which show substantial individual differences in depth and number, and are related to hippocampal volume and CA1 neuronal count (Adler et al., 2018; Simic, Kostovic, Winblad, & Bogdanovic, 1997; Figures 1 and 2).

**FIGURE 3:**
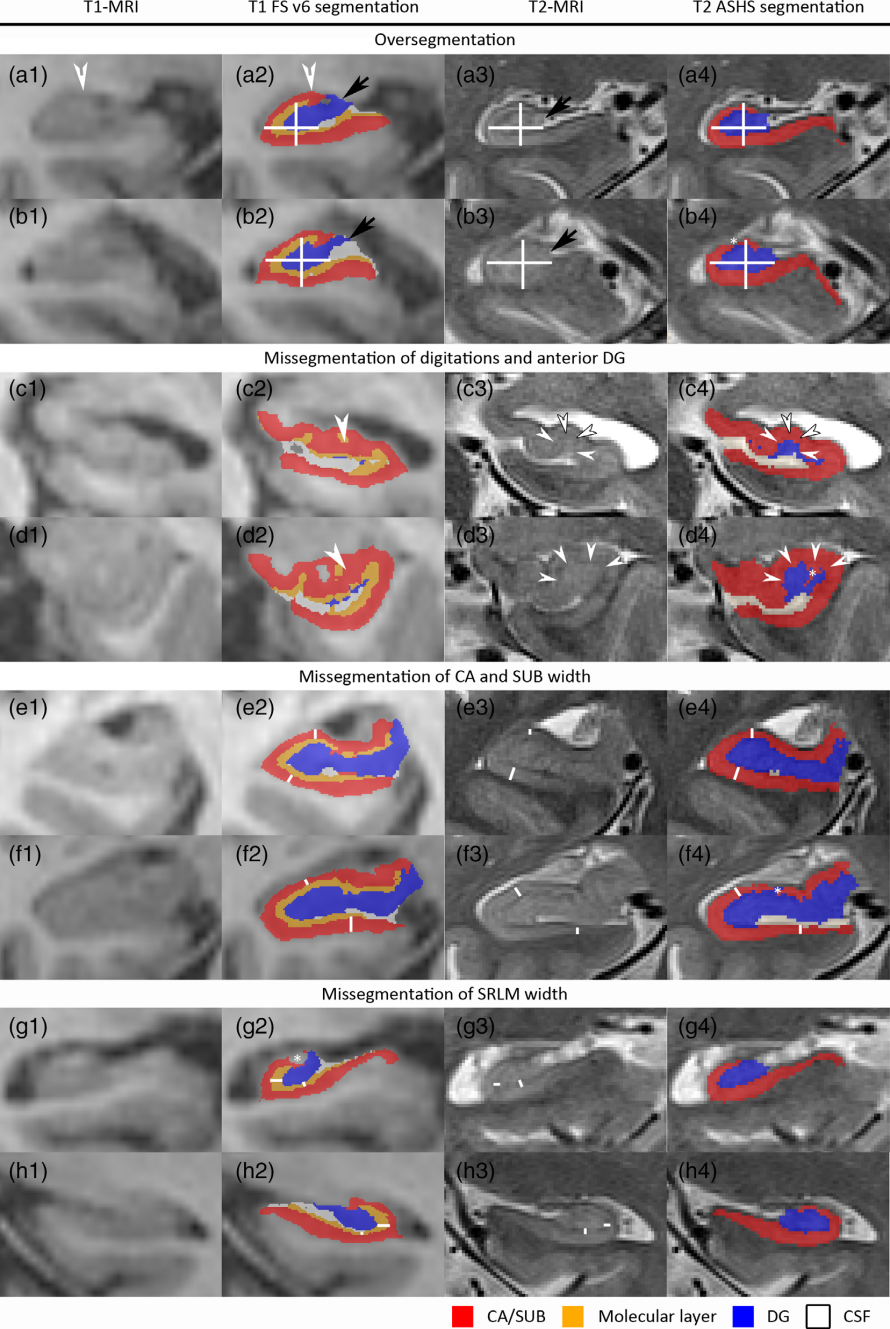
Qualitative within‐subject comparison of automatic segmentation of hippocampal subfields on T_1_‐ and T_2_‐weighted MRI. To make a fair comparison, we only selected automatic methods. Specifically, we selected the two methods that are most commonly used for T_1_‐ and T_2_‐weighted MRI respectively. We selected FreeSurfer (FS) version 6.0 (Iglesias et al., 2015) for T_1_‐weighted MRI and Automatic Segmentation for Hippocampal Subfields (ASHS) (Yushkevich et al., 2015) for T_2_‐weighted MRI. MRI scans from two patients with Mild Cognitive Impairment and two cognitively normal older adults were selected. The T_1_‐weighted MRI was registered to the T_2_‐weighted MRI using rigid registration, as done by the ASHS pipeline (Xie et al., 2019). Specifically, the T_2_‐weighted MRI was up‐sampled to isotropic resolution and rigid registration in the “Greedy” tool (https://github.com/pyushkevich/greedy) was performed using the mutual information image similarity metric with multi‐resolution optimization (×4, ×2, and ×1 subsampling). The T_2_‐weighted MRI image was further resampled to 0.2 × 0.2 × 0.2 mm^3^ resolution and the T_1_‐weighted MRI and its segmentation were resliced into the 0.2 × 0.2 × 0.2 mm^3^ up‐sampled T_2_‐weighted MRI space, thus preserving as much of the detail in the segmentation as possible. The quality of image registration was verified in ITK‐SNAP. Moreover, grids are displayed so the registration quality can be checked. To facilitate the comparison of the two different methods, we collapsed the labels for the different subiculum and cornu ammonis (CA) regions into one label (red) and the dentate gyrus (DG) and granular cell layer into another label (blue). We kept the label for the molecular layer (orange) generated by FS (note that this label is often referred to as stratum radiatum lacunosum moleculare or SRLM). For ASHS, a general agreement can be observed between the features in the T_1_‐weighted MRI and the segmentation boundaries; however, there is less agreement for FS. Both the FS and ASHS segmentations contained errors, which are inherent to automatic segmentations. In this figure, we display four typical FS segmentation errors, but also point out errors in the ASHS segmentations (b4, d4, f4). Given the size of the FS segmentation errors, it is unlikely that partial volume effects on T_2_‐weighted MRI and small registration errors can fully explain the errors observed on T_1_‐weighted MRI. While the observed FS errors may seem small, hippocampal subfields are very thin and small errors can therefore have a large impact on the measurements of these granular regions. Figures a1‐a4 and b1–b4 exemplify over‐segmentation of the hippocampus, in general, where the outer boundary of the segmentation does not match that of the hippocampus on MRI. The black arrows (a2–3, b2–3) indicate the over‐segmentation of the DG, such that cerebrospinal fluid and the fimbria is included in the DG label, even though there is a separate fimbria label in FS, which is not displayed in these images. The white arrows (a1–2) indicate the over‐segmentation of the CA (likely CA2 and CA3), including cerebrospinal fluid and alveus in the CA label, even though there is a separate label for the alveus which is not displayed in these images. Over‐segmentation is further exemplified by the white crosses showing the width and height of the hippocampus, which is fairly well‐matched by the ASHS segmentation but not by the FS segmentation. An asterisk indicates a small segmentation error for the ASHS segmentation. The second block shows the mis‐segmentation of the anterior DG and the digitations. The SRLM or dark band is indicated by white arrows in c3 and d3; this indicates the border of the DG (blue) with surrounding subfields. As shown in c2 and d2, the FS segmentation does not match the anatomy as hardly any DG is present and the yellow label does not match the SRLM (hypointense on the T_2_‐weighted MRI). This means that DG is erroneously included in the molecular and CA labels. ASHS, on the other hand, shows a more accurate segmentation of the DG, although an error (*) can be observed in d4. The third block shows the mis‐segmentation of CA and the subiculum. The width of CA and the subiculum is indicated by white lines in several places on the T_2_‐weighted MRI (e3 and f3) and is also marked in approximately the same location in the ASHS and FS segmentation. As can be observed in e2 and f2, the width of CA/subiculum is not correctly picked up by FS. FS also shows inconsistencies in the width measurement, such that this region is sometimes thinner and sometimes thicker than the actual width. Although the ASHS segmentation (e4 and f4) is not perfectly matched to the MRI, it is fairly close and more consistent with the actual width. Note that if the width of the CA/subiculum is not correctly estimated, the volume measurements will also be off. The fourth block shows the mis‐segmentation of the SRLM width. The SRLM width is indicated by white lines on the T_2_‐weighted MRI and approximately the same location in the FS segmentation. While the orange label of the molecular layer in the FS segmentation sometimes approximates the actual SRLM width, in other places it is much thicker or thinner. As the thickness of SRLM is not correctly estimated, the volume measurements will also be inaccurate. Moreover, note a small FS segmentation error in g2

Because the SLRM is a thin structure, ~1 mm^3^ isotropic scans are insufficient for visualizing this important landmark. A recent postmortem study indicated that the hypointense band in 0.2 × 0.2 × 0.2 mm^3^ T_2_‐weighted MRI corresponding to SRLM is ~0.87 mm thick (range 0.77–1.05 mm) in nondemented older adults and 0.65 mm (range 0.52–0.88 mm) in patients with AD (Adler et al., 2018). To the best of our knowledge, no information is available on other populations or age groups at the time of this writing. Thus, visualizing SRLM in vivo requires MRI with high in‐plane resolution. Consistent visualization of this landmark is crucial for separating the DG from the other subfields, even if SRLM is not segmented and measured as a separate structure. Given that the SRLM is thinner than a single 1 mm voxel, and thus can rarely be visualized on ~1 mm^3^ isotropic MRI scans, it is unlikely that these scans can yield hippocampal subfield volume estimate by either manual or automatic segmentation. This difficulty is illustrated in Figure 2, which shows within‐subject comparisons of high‐resolution T_2_‐weighted MRI and 1 mm^3^ isotropic T_1_‐weighted MRI scans, and in Figure 3, which demonstrates a qualitative within‐subject comparison of an automatic segmentation method applied to both T_1_‐weighted scans and high resolution T_2_‐weighted scans. Finally, Figure S1 shows an automatic segmentation of a 1 mm^3^ isotropic T_1_‐weighted MRI scan compared to manual segmentation of a high resolution proton density weighted image.

## 3 REVIEW OF VALIDATION OF SUBFIELD SEGMENTATION ON ~1 MM^3^ ISOTROPIC IMAGES

The preferred validation, or “the gold standard,” of automatic segmentation would be a comparison with annotated histology sections. This would require matched in vivo and ex vivo imaging data with hippocampal subfield boundaries traced on ex vivo histology, all within the same subject. Such data sets are exceedingly rare (e.g., only 15, sometimes partial, specimens were available in Goubran et al., 2016 and two in Wisse et al., 2016). It is therefore common to validate automatic segmentation against expert manual segmentation—the “bronze standard.” Such validation typically compares the accuracy of the automatic segmentation relative to reliable manual segmentation and then compares the resulting value(s) to the inter‐rater and intra‐rater reliability of manual segmentation. When errors made by an algorithm relative to reliable manual segmentation are statistically equivalent to the disagreements made between different expert raters, it can be argued that the algorithm is an acceptable stand‐in for manual segmentation. In other words, if the reliability of an automated algorithm compared with a manual rater is similar to the reliability of two manual raters, the algorithm can be considered as an acceptable stand‐in for manual segmentation. See Box 1 for more information on the terminology used here and in the sections below.

Box 1: **Terminology**

*Accuracy*: Accuracy is the agreement of a measurement relative to an external standard.

*Reliability*: Reliability is the internal consistency of a measure, which can be evaluated by the agreement between repeated measures (e.g., between raters or multiple assessments). This is similar to precision.

*Construct validity or validity*: Construct validity is the degree to which a test measures what it purports to be measuring. In the case of hippocampal subfield segmentation, construct validity refers to the degree to which the segmentation protocol correctly reflects known hippocampal subfield anatomy.

*Convergent validity*: Convergent validity is a form of construct validity. It refers to the degree to which two measures of constructs that theoretically should be related, are in fact related.

*Face validity*: Face validity is the degree to which a procedure appears effective in terms of its stated aims. In application to hippocampal subfield segmentation, it may correspond to the qualitative similarities between a segmentation on in vivo MRI as compared to an atlas image with histological labeling.

*Prior*: A prior in the context of FreeSurfer subfield segmentation is prior knowledge of hippocampal subfield anatomy obtained by combining anatomical annotations of ex vivo MRI scans in the FreeSurfer 6.0 atlas. It can be thought of as the “average” hippocampal subfield anatomy along with statistical information describing how individual anatomies deviate from the average. This information is captured relative to the overall shape of the hippocampus. This statistical representation of anatomy is coupled with information on MRI appearance of hippocampal subfields from the ex vivo atlas (e.g., SRLM voxels have lighter appearance than CA1 voxels). When segmenting a new MRI scan, FreeSurfer uses information both from this prior model and from the MRI scan being segmented to determine how to deform the atlas to match intensity features in the new MRI. However, when intensity features in the new MRI are not informative (e.g., when the intensity values within the hippocampus are largely homogeneous), it is unlikely that these features influence the deformation of the atlas, and so the final segmentation is likely to be driven by atlas information (i.e., the prior) alone.

We next discuss several different automatic segmentation methods. In the first section, we discuss the most commonly used method for automatic segmentation of hippocampal subfields, implemented in FreeSurfer software (Iglesias et al., 2015; Van Leemput et al., 2009). This method has not been validated against manual segmentation as applied to ~1mm^3^ isotropic T1‐weighted images. In the second section, we discuss the validation of two other automatic methods for hippocampal subfield segmentation.

### 3.1 Hippocampal subfield segmentation in the different FreeSurfer versions

The original van Leemput et al. method implemented in FreeSurfer 5.3 was evaluated against manual segmentation in high‐resolution MRI scans (0.38 × 0.38 × 0.38 mm^3^) (Van Leemput et al., 2009), but only in a few slices of the hippocampal body and not against manual segmentations of the same protocol in lower‐resolution ~1 mm^3^ isotropic data, to which it is commonly applied. Although FreeSurfer 5.3 has been deprecated, as stated on the software website, (https://surfer.nmr.mgh.harvard.edu/fswiki/HippocampalSubfieldSegmentation) and subfield segmentation performed with this version has been criticized for low construct validity (Box 1) of the segmentation protocol (de Flores et al., 2015; Wisse, Biessels, & Geerlings, 2014), FreeSurfer 5.3 is nonetheless still being used by multiple research groups to segment hippocampal subfields (Duan et al., 2020; Izzo, Andreassen, Westlye, & van der Meer, 2020; Takaishi et al., 2020).

The hippocampal subfield segmentation module in FreeSurfer 6.0 (Iglesias et al., 2015) uses an ex vivo atlas derived from 15 ex vivo MRI scans of the hippocampus and accompanying detailed annotations of 13 hippocampal subfields, which improved the face validity of this version over version 5.3. This, together with the large number of experiments performed in the Iglesias et al. paper (2015), may lead readers to conclude that FreeSurfer 6.0 has been completely validated. However, the segmentations provided by FreeSurfer 6.0 on in vivo MRI scans have not been validated against histological annotations in the same subjects (gold standard), nor have these segmentations been validated against manual segmentation on the same MRI scans using the FreeSurfer 6.0 13‐label protocol (bronze standard). As such, the validity of these algorithms when applied to ~1 mm^3^ isotropic T_1_‐weighted scans remains unknown.

In the absence of extensive validation, researchers should not assume that automatic methods produce anatomically accurate segmentations on images that lack sufficient anatomical detail that would allow manual segmentation. While it is theoretically possible that an automatic algorithm might detect and exploit some anatomical features in ~1 mm^3^ isotropic MRI scans that a trained expert cannot, a more likely explanation is that automatic methods fill in missing information (e.g., SRLM) by using anatomical priors (Box 1). The creators of Freesurfer's automatic tool acknowledged this on their website: *“When segmenting 1 mm scans*, *the position of the internal boundaries between the hippocampal substructures largely relies on prior knowledge acquired from our ex vivo training data and summarized in our statistical atlas*.” (Iglesias, 2019). The resulting interpolation of subfields based on priors and visible features, such as the overall hippocampus boundary, is likely to ignore individual variation in hippocampal subfield anatomy, such as variation in the ratio of DG and CA thickness or variation in the number and location of digitations in the hippocampal head (see Figure 3).

The authors of this commentary therefore express concerns that automatic subfield volumetric measurements generated by FreeSurfer using ~1 mm^3^ isotropic images—currently the most widely used method for subfield segmentation—likely do not capture variation in subfield volumes per se, but rather act as a proxy of total volume. They are, therefore, likely unable to capture specific variability in anatomical features, for example, selective thinning in some regions or patterns of digitations in the hippocampal head (see, for example, findings by Elman et al. in support of this statement (Elman et al., 2018). The limitations of automatic subfield segmentation on ~1 mm^3^ isotropic T_1_‐weighted images have been pointed out previously (de Flores, La Joie, Landeau, et al., 2015; Wisse et al., 2014), and similar limitations would, of course, apply to volume estimates from manual segmentation of images of the same resolution. Moreover, the creators of the FreeSurfer 6.0 subfield segmentation algorithm (Iglesias et al., 2015) stated in their paper that the use of subfield segmentation based on ~1 mm^3^ isotropic T_1_‐weighted images was better suited “*as seed and target regions in functional and diffusion MRI studies*,” and cautioned against the interpretation of subfield volumes in quantitative analyses (Iglesias et al., 2015). These concerns also hold for the subfield segmentation algorithm recently introduced in FreeSurfer version 7.0 as well as future versions that provide similar automatic subfield volumetric measurements on ~1 mm^3^ isotropic images.

The findings presented in Box 2 support our concerns regarding subfield segmentation on ~1 mm^3^ isotropic images for the estimation of volumes. In short, we compared FreeSurfer 6.0 segmentation of 1 mm^3^ isotropic T_1_‐weighted images to manual segmentation of high‐resolution proton density‐weighted images with respect to the ability to capture subfield volume associations with MCI and AD, similar to a previous comparison of Freesurfer 5.3 with manual segmentations (de Flores, La Joie, Landeau, et al., 2015). The hippocampal subfield volumes generated by FreeSurfer 6.0 failed to reveal significant AD‐related differences in the expected subfield, CA1, which is the first region in the hippocampus to accrue neurofibrillary tangle pathology (Braak & Braak, 1991). In contrast, smaller CA1 volume in β‐amyloid positive MCI patients compared to controls was found in the manually segmented proton density dataset in the same subjects, in line with multiple in vivo MRI studies (reviewed by de Flores, La Joie, & Chetelat, 2015). Although these types of comparisons cannot replace the comparison of automated or manual segmentations against histology annotations in the same subject, we believe that a segmentation method—manual or automated—that is insensitive to clinically relevant changes in diseases will have limited utility.

Box 2: **FreeSurfer 6.0 comparison with manual segmentation in controls, MCI and AD patients, as in De Flores et al. (de Flores, La Joie, Landeau, et al., 2015)**

In this box, we aim to compare hippocampal subfield volumes between older adults, patients with Mild Cognitive Impairment (MCI) and Alzheimer's disease (AD). Similar to De Flores et al. (de Flores, La Joie, Landeau, et al., 2015), we aim to compare the performance of automatic segmentations implemented in FreeSurfer (FS) 6.0 (Iglesias et al., 2015) on 1 × 1 × 1 mm^3^ T_1_‐weighted MRI and manual segmentations on high‐resolution 0.4 × 0.4 × 2 mm^3^ (2 mm gap) proton density weighted images (which have similar contrast as T_2_‐weighted images). Twenty‐eight older adults (mean age: 70.3 ± 6.5 years; 46.4% men; education: 12.4 ± 4.1 years), 9 β‐amyloid positive (A+, cut off based on 3 standard deviations from a young control group) patients with MCI (mean age: 70.0 ± 5.5 years; 42.9% men; education: 10.7 ± 4.8 years) and 13 A+ patients with AD (mean age: 65.2 ± 10.3 years; 30.8% men; education: 10.8 ± 4.3 years) were included. Note that a smaller number of subjects was included than in de Flores, La Joie, and Chetelat (2015) because the FreeSurfer 6.0 pipeline failed in some subjects and only A+ patients were included. All manual segmentations were performed by author RLJ. The intraclass correlation coefficient was 0.94 for cornu ammonis (CA) 1, 0.89 for subiculum and 0.94 for CA2/3/dentate gyrus (La Joie et al., 2010). All subfield volumes were corrected for intracranial volume and analyses of covariance were performed, including age, gender, and education as covariates. Based on Braak staging of neurofibrillary tangle pathology (Braak & Braak, 1991), which is closely related to neurodegeneration (Bobinski et al., 1997; Fukutani et al., 1995), the earliest and strongest volume loss is expected in CA1, then the subiculum, while the other CA regions and the dentate gyrus are expected to be affected in later stages.

The Table 1 demonstrates that segmentations generated by FS 6.0 do not reflect the expected pattern of atrophy, with only CA2/3/4/dentate gyrus surviving Bonferroni correction in the comparison between A+ MCI and older adults, and with all regions showing a similar effect when comparing older adults with A+ patients with AD. Splitting up the subfields in the different labels provided by FS 6.0 does not change the results (see Table S1). Conversely, using manual segmentation, CA1 was found as the most atrophied region in A+ MCI patients as compared to older adults, whereas, as expected, both CA1 and subiculum were significantly smaller compared to A+ AD. CA2/3/4/dentate gyrus showed the weakest effect size when comparing older adults to both A+ MCI and A+ AD patients. Note that, based on a comparison of 95% CI, the effect sizes are equivalent, except in CA2/3/4/dentate gyrus comparing A+ MCI patients and older adults. See Figure S1 for a comparison of the manual and automatic segmentation.

These results indicate that FreeSurfer 6.0 hippocampal subfield volumes measured on standard T_1_‐weighted images do not capture the expected pattern of atrophy over the course of AD, while an appropriate manual segmentation performed on high resolution images in the same subjects does. Note that this comparison does not allow any conclusions about the application of FreeSurfer 6.0 package to high resolution T_2_‐weighted images.

### 3.2 Validation of other automatic methods against manual segmentation

In this section, we discuss two methods that developed an approach to compare automatic segmentations of hippocampal subfields against manual segmentations on ~1 mm^3^ isotropic images. Two studies (Caldairou et al., 2016; Pipitone et al., 2014) down‐sampled manual subfield segmentations obtained on high resolution (Caldairou: 0.6 × 0.6 × 0.6 mm^3^; Pipitone: 0.3 × 0.3 × 0.3 mm^3^) T_1_ and T_2_‐weighted images to ~1 mm^3^ isotropic resolution (Caldairou: 1x1x1 mm^3^; Pipitone: 0.9x0.9x0.9 mm^3^) T_1_‐weighted MRI scans from the same subjects. The down‐sampled data from the same subjects served to evaluate the performance of the automatic segmentation algorithms. Although the Dice Similarity Coefficient (DSC) values presented by Caldairou et al. (2016) exceeded 0.80, they were considerably lower, that is, between 0.41–0.65, in the Pipitone et al. (2014) paper. These lower DSC values may be due to the higher complexity of the segmentation protocol (Pipitone et al., 2014; Winterburn et al., 2013), the smaller number of atlases, and the wider age range of subjects included in the atlas set. The explanation for the higher DSC values in the Caldairou et al. (2016) paper could be two‐fold. One possibility is that there is enough subtle signal information to infer the location of DG/CA/subiculum from 1 mm^3^ T_1_‐weighted MRI (these are larger, and geometrically less complex labels than in the protocol (Winterburn et al., 2013) used in Pipitone et al. (2014) paper), which would mean that it is acceptable to use their method in ~1 mm^3^ isotropic MRI. The other possibility is that the segmentation is driven by a shape prior, given that the authors discuss using a strong prior, and the location of the subfields is sufficiently predictable from the shape prior, for example, because this is a young population, to get the high DSC values. Additionally, the high DSC values may be partially explained by the fact that the included subfield or subfield groups were relatively large in size, a factor that positively affects DSC values. Indeed, reported correlation coefficients were considerably lower in the Caldairou et al. study: 0.28–0.64. We would like to stress that this evaluation speaks to the consistency of this specific method in this cohort but does not generalize to provide evidence that all automatic methods can accurately measure subfields in ~1 mm^3^ isotropic images, nor that the same method will perform adequately in other populations. In fact, the relatively low correlation coefficients in the Caldairou et al. (2016) and the relatively low DSC values in the Pipitone et al. (2014) warrant further caution toward obtaining hippocampal subfield volumes from 1 mm^3^ isotropic MRI scans. Finally, applying one of these methods to ~1 mm^3^ isotropic MRI scans from other populations will not allow for careful assessment of the quality of the segmentations given the limited detail available in these images.

## 4 CONCERNS REGARDING ANOTHER VALIDATION APPROACH FOR SUBFIELD SEGMENTATION ON ~1 MM^3^ ISOTROPIC MRI

Finally, we caution against validating FreeSurfer 6.0 ~ 1 mm^3^ isotropic T_1_‐weighted MRI (FS‐T_1_) segmentations against Freesurfer 6.0 applied on a combination of T_1_‐weighted MRI and high‐resolution T_2_‐weighted MRI (FS‐T_1_T_2_) as a standard. This comparison offers little information on validity, as FS‐T_1_T_2_ has not yet been validated against manual segmentation or histology. Moreover, the validation of FS‐T_1_ against FS‐T_1_T_2_ is inherently biased, given that segmentation of both FS‐T_1_ and FS‐T_1_T_2_ is based on some combination of intensity features and shape priors. To illustrate this point, suppose that in both cases, 100% of segmentation information came from shape priors (Box 1). One would then observe a perfect correlation between FS‐T_1_ and FS‐T_1_T_2_. Measuring the correlation between FS‐T_1_ and FS‐T_1_T_2_ therefore has the unfortunate side effect of measuring the strength of the priors. A high correlation could either be due to T_1_ and T_1_ + T_2_ providing similar information for segmentation, or due to a strong reliance on a prior (e.g., that the hippocampus has a certain internal structure, that each structure has a typical shape and volume, and that by warping the outer surface, the inner structures are similarly warped). The inability to disambiguate the two factors driving the metric make it flawed for evaluating FS‐T_1_ anatomical validity. Relating to this point, the use of the FreeSurfer subfield package has in the past been justified by high test–retest reliability, but test–retest reliability does not speak to construct validity. In contrast, high test–retest reliability likely shows the strength of the prior. Note that these concerns also hold for FreeSurfer version 7.0 and any similar future versions.

## 5 ALTERNATIVE METHODS TO OBTAIN GRANULAR STRUCTURAL MEASURES OF THE HIPPOCAMPUS USING ~1 MM^3^ ISOTROPIC MRI

We are unfortunately not able to provide alternatives for obtaining hippocampal subfield volumetric measures from ~1 mm^3^ isotropic MRI, given the above stated limitations of these kind of images. However, alternative approaches exist for obtaining more granular measures than those available via whole hippocampal volume using ~1 mm^3^ isotropic MRI. For example, researchers have divided the hippocampus along its long axis (head, body and tail; e.g., Bernasconi et al., 2003; Chen, Chuah, Sim, & Chee, 2010; Daugherty, Yu, Flinn, & Ofen, 2015; Malykhin, Carter, Seres, & Coupland, 2010) and have also used surface deformation‐based methods (e.g., Apostolova et al., 2012; Wang et al., 2006). While these methods cannot make inferences about the inner subfields, they can nonetheless provide more granular and highly relevant measures of hippocampal structure that may be more sensitive to functional and clinical correlates than whole hippocampal volume.

## 6 CONCLUSION

The interest in MRI‐based hippocampal subfield research has significantly increased in recent years due to availability of public‐domain datasets, such as the ADNI (Weiner et al., 2017) and human connectome datasets (Van Essen et al., 2013), as well as by publicly available automatic tools, such as FreeSurfer (Iglesias et al., 2015; Van Leemput et al., 2009), ASHS (Yushkevich et al., 2015), and MAGeT (Pipitone et al., 2014; Winterburn et al., 2013). Although these developments enable large‐scale subfield analyses, it is important to remain cautious regarding the increasing application of automatic segmentation methods to inappropriate data sets (e.g., ~1 mm^3^ isotropic T_1_‐weighted MRI scans) for several reasons. First, the resolution of these images is insufficient for visualizing the inner structures of the hippocampus, particularly the SRLM, that are crucial for either manual or automatic subfield segmentation. Second, automatic subfield segmentation on ~1 mm^3^ isotropic images has not been validated against manual segmentation for some methods, including FreeSurfer (Iglesias et al., 2015; Van Leemput et al., 2009), the most commonly used approach. We are therefore concerned that subfield volumetric data from ~1 mm^3^ isotropic MRI scans are not capturing subfield volumes as intended, but rather represent a proxy of total volume and are not able to capture specific variability in anatomical features. It should be noted that there are some methods that have validated automatic hippocampal subfield segmentations against manual segmentations down‐sampled to ~1 mm^3^ isotropic MRI scans (Caldairou et al., 2016; Pipitone et al., 2014). However, the results require careful scrutiny before considering the application of such methods to a particular data set.

Although our concerns are partly based on reasoning and formal comparisons of images acquired with different weighting (T_1_ vs. T_2_) and resolution, we believe the arguments outlined in this commentary are strong enough to warrant caution against hippocampal subfield segmentation on ~1 mm^3^ isotropic MRI scans, a caution supported by other research groups (e.g., Elman et al., 2018; Giuliano et al., 2017; Iglesias et al., 2015). We recommend that future studies further compare hippocampal subfield segmentation on ~1 mm^3^ isotropic MRI scans with higher resolution MRI scans. We believe that such studies will provide additional data highlighting the need for caution when attempting to segment hippocampal subfields on ~1 mm^3^ isotropic images and replicate some of the previous comparison papers (de Flores, La Joie, Landeau, et al., 2015; Mueller et al., 2018).

## CONFLICT OF INTERESTS

None of the authors has any disclosures.

## Supporting information


**Appendix** **S1.** Supporting Information.

**Appendix** **1.**

## Data Availability

Data sharing is not applicable to this article as no new data were created or analyzed in this study.
