# Supplementary material for: Hippocampal subfield volumetry from structural isotropic 1 mm3 MRI scans: A note of caution
Source: Hum Brain Mapp. 2020 Oct 15;42(2):539–50. doi: 10.1002/hbm.25234 (PMC7775994; doi:10.1002/hbm.25234)

**Supplementary table 1.** Differences in hippocampal subfield volumes in older adults, patients with MCI and patients with AD using automated FreeSurfer 6.0 segmentation of T_1_-weighted MRI. Bold font indicates significance after Bonferroni correction (0.05/39)

|  | **Older adults vs MCI** | | **Older adults vs AD** | | **MCI vs AD** | |
| --- | --- | --- | --- | --- | --- | --- |
| **FS6-T1** | **Effect size (95% CI)** | **p-value** | **Effect size (95% CI)** | **p-value** | **Effect size (95% CI)** | **p-value** |
| CA1 | 1.19 (0.28 ; 2.10) | 0.02 | 2.35 (1.49 ; 3.21) | **<0.001** | 0.97 (0.07 ; 2.00) | 0.01 |
| CA2/3 | 1.14 (0.24 ; 2.04 ) | 0.01 | 1.71 (0.93 ; 2.48 ) | **<0.001** | 0.63 (0.37 ; -1.64 ) | 0.27 |
| CA4 | 1.82 (0.85 ; 2.79 ) | **<0.001** | 2.25 (1.4 ; 3.09 ) | **<0.001** | 0.36 (0.64 ; 1.35) | 0.35 |
| HATA | 0.43 (-0.44 ; 1.29 ) | 0.72 | 2.28 (1.43 ; 3.13 ) | **<0.001** | 1.29 (0.22 ; 2.36) | 0.001 |
| Molecular layer | 1.53 (0.59 ; 2.47 ) | 0.002 | 2.76 (1.84 ; 3.67 ) | **<0.001** | 0.94 (0.09 ; 1.97) | 0.004 |
| GC–DG | 1.66 (0.71 ; 2.61 ) | **<0.001** | 2.1 (1.27 ; 2.92 ) | **<0.001** | 0.40 (0.59 ; 1.39) | 0.22 |
| Subiculum | 1.01 (0.12 ; 1.9 ) | 0.04 | 2.51 (1.63 ; 3.4 ) | **<0.001** | 1.42 (0.33 ; 2.51) | **<0.001** |
| Presubiculum | 1.03 (0.13 ; 1.92 ) | 0.04 | 2.43 (1.56 ; 3.3 ) | **<0.001** | 1.35 (0.27 ; 2.43) | 0.005 |
| Parasubiculum | 0.32 (-0.54 ; 1.18 ) | 0.62 | 1.68 (0.91 ; 2.46 ) | **<0.001** | 1.29 (0.22 ; 2.37) | 0.11 |
| Fimbria | -0.03 (-0.89 ; 0.83 ) | 0.93 | 0.12 (-0.55 ; 0.8 ) | 0.72 | 0.18 (0.8 ; 1.17) | 0.64 |
| Tail | 1.86 (0.89 ; 2.83 ) | **<0.001** | 2.44 (1.57 ; 3.31 ) | **<0.001** | 0.58 (0.43 ; 1.58) | 0.27 |
| Hippocampal fissure | 0.00 (-0.86 ; 0.86 ) | 0.99 | 0.42 (-0.26 ; 1.11 ) | 0.13 | 0.42 (0.58 ; 1.41) | 0.35 |
| Whole Hippocampus | 1.56 (0.62 ; 2.50) | 0.002 | 2.70 (1.79 ; 3.61) | **<0.001** | 0.93 (0.10 ; 1.96) | 0.01 |

AD=Alzheimer’s disease, CA=cornu ammonis, GC-DG=granular cell layer of the dentate gyrus, HATA=hippocampal amygdala transition area, MCI=mild cognitive impairment

**Supplementary Figure 1.** A comparison of manual segmentations performed on 0.4x0.4x2 mm^3^ (2 mm gap) proton density weighted images and FreeSurfer 6.0 segmentations using 1x1x1 mm^3^ T_1_-weighted images in an older adult and a patient with Alzheimer’s disease. Additionally, a comparison of the upsampled high-resolution segmentation as generated by FreeSurfer 6.0 and the same segmentation at the original resolution is shown. In the right panel it becomes clear that certain labels included in the FreeSurfer algorithm are smaller than the original voxel size. CA=cornu ammonis; DG=dentate gyrus; GC=granular cell layer; HATA=hippocampal amygdala transition area; ML=molecular layer; SUB=subiculum


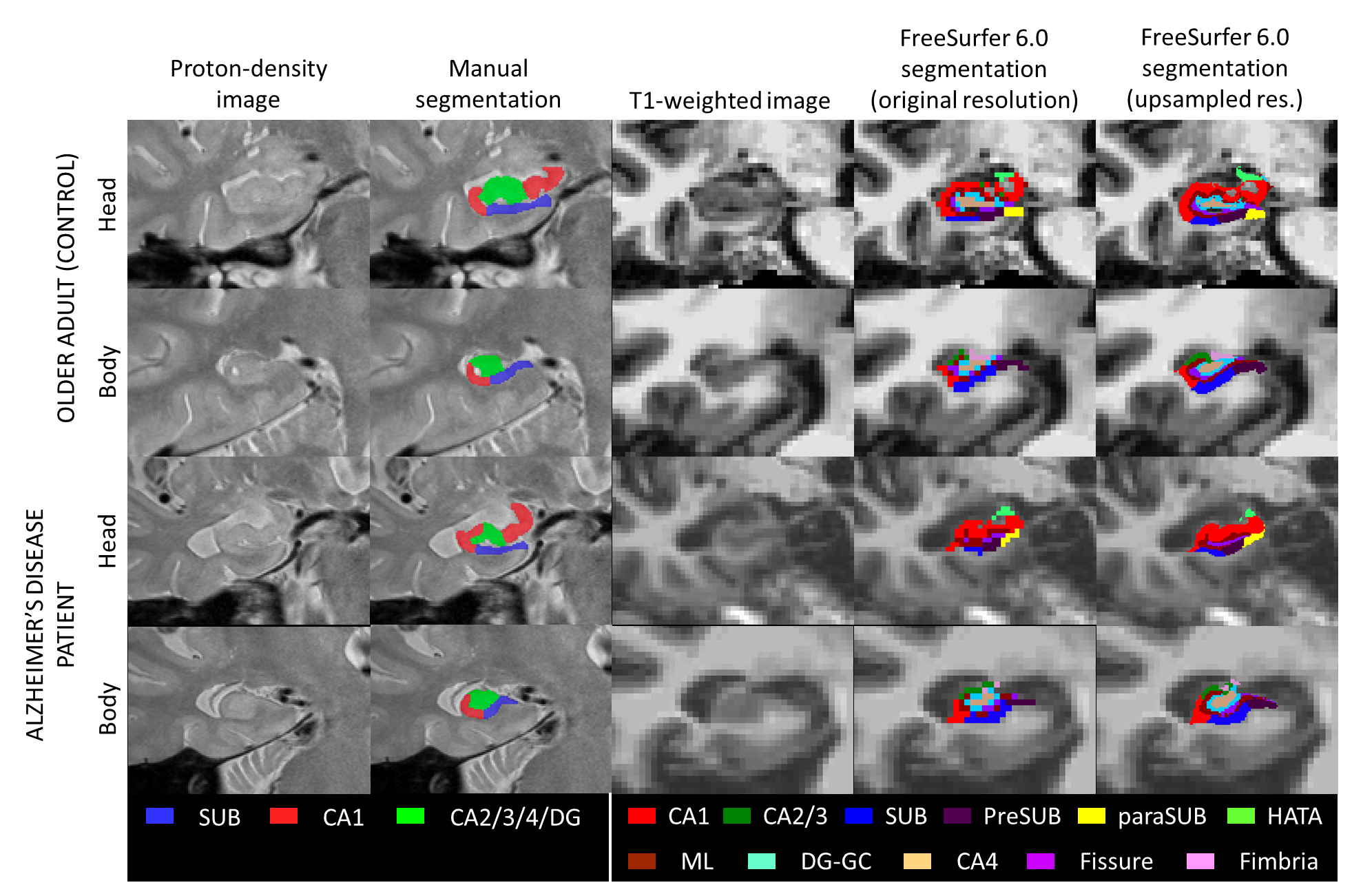

Supplement: Supplementary file 1 — Appendix S1. Supporting Information. [file HBM-42-539-s001.docx]
