# Supplementary material for: Hippocampal subfield volumetry from structural isotropic 1 mm3 MRI scans: A note of caution
Source: Hum Brain Mapp. 2020 Oct 15;42(2):539–50. doi: 10.1002/hbm.25234 (PMC7775994; doi:10.1002/hbm.25234)
Supplement: Supplementary file 2 — Appendix 1. [file HBM-42-539-s002.docx]

**Publications performing hippocampal subfield segmentation on ~1 mm^3^ isotropic MRI**

[1] Al-Amin M, Zinchenko A, Geyer T. Hippocampal subfield volume changes in subtypes of attention deficit hyperactivity disorder. *Brain Res* 2018;**1685**.

[2] Aslaksen PM, Bystad MK, Ørbo MC*, et al*. The relation of hippocampal subfield volumes to verbal episodic memory measured by the California Verbal Learning Test II in healthy adults. *Behav Brain Res* 2018;.

[3] Averill CL, Satodiya RM, Scott JC*, et al*. Posttraumatic Stress Disorder and Depression Symptom Severities Are Differentially Associated With Hippocampal Subfield Volume Loss in Combat Veterans. *Chronic Stress* 2017;**1**.

[4] Baglivo V, Cao B, Mwangi B*, et al*. Hippocampal subfield volumes in patients with first-episode psychosis. *Schizophr Bull* 2017;**44**(3).

[5] Beale C, Broyd SJ, Chye Y*, et al*. Prolonged Cannabidiol Treatment Effects on Hippocampal Subfield Volumes in Current Cannabis Users. *Cannabis and cannabinoid research* 2018;**3**(1).

[6] Bocchetta M, Iglesias JE, Scelsi MA*, et al*. Hippocampal subfield volumetry: differential pattern of atrophy in different forms of genetic frontotemporal dementia. *J Alzheimer's Dis* 2018;(Preprint).

[7] Bódi N, Polgár A, Kiss E*, et al*. Reduced volumes of the CA1 and CA4-dentate gyrus hippocampal subfields in systemic lupus erythematosus. *Lupus* 2017;**26**(13).

[8] Bookheimer SY, Salat DH, Terpstra M*, et al*. The lifespan Human Connectome Project in aging: An overview. *Neuroimage* 2018;.

[9] Brody GH, Gray JC, Yu T*, et al*. Protective prevention effects on the association of poverty with brain development. *JAMA pediatrics* 2017;**171**(1).

[10] Caldwell JZ, Berg J, Shan G*, et al*. Sex Moderates the Impact of Diagnosis and Amyloid PET Positivity on Hippocampal Subfield Volume. *J Alzheimer's Dis* 2018;(Preprint).

[11] Cao B, Passos IC, Mwangi B*, et al*. Hippocampal subfield volumes in mood disorders. *Mol Psychiatry* 2017;**22**(9).

[12] Cao B, Luo Q, Fu Y*, et al*. Predicting individual responses to the electroconvulsive therapy with hippocampal subfield volumes in major depression disorder. *Scientific reports* 2018;**8**(1).

[13] Chen Z, Chen X, Liu M*, et al*. Lower hippocampal subfields volume in relation to anxiety in medication-overuse headache. *Molecular pain* 2018;**14**.

[14] Chen LW, Sun D, Davis SL*, et al*. Smaller Hippocampal CA-1 Subfield Volume in Posttraumatic Stress Disorder. *bioRxiv* 2018;.

[15] Doolin K, Allers KA, Pleiner S*, et al*. Altered tryptophan catabolite concentrations in major depressive disorder and associated changes in hippocampal subfield volumes. *Psychoneuroendocrinology* 2018;**95**.

[16] Evans TE, Adams HH, Licher S*, et al*. Subregional volumes of the hippocampus in relation to cognitive function and risk of dementia. *Neuroimage* 2018;**178**.

[17] Foo H, Mak E, Chander RJ*, et al*. Associations of hippocampal subfields in the progression of cognitive decline related to Parkinson's disease. *NeuroImage: Clinical* 2017;**14**.

[18] Greenspan KS, Arakelian CR, van Erp TG. Heritability of hippocampal formation sub-region volumes. *Journal of neurology and neuroscience* 2016;**7**(6).

[19] Györfi O, Nagy H, Bokor M*, et al*. reduced ca2–ca3 hippocampal subfield Volume is related to Depression and normalized by L-DOPa in newly Diagnosed Parkinson’s Disease. *Frontiers in neurology* 2017;**8**.

[20] Haukvik UK, Tamnes CK, Söderman E*, et al*. Neuroimaging hippocampal subfields in schizophrenia and bipolar disorder: A systematic review and meta-analysis. *J Psychiatr Res* 2018;.

[21] Hibar DP, Adams HH, Jahanshad N*, et al*. Novel genetic loci associated with hippocampal volume. *Nature communications* 2017;**8**.

[22] Ho NF, Iglesias JE, Sum MY*, et al*. Progression from selective to general involvement of hippocampal subfields in schizophrenia. *Mol Psychiatry* 2017;**22**(1).

[23] Ho NF, Holt DJ, Cheung M*, et al*. Progressive decline in hippocampal CA1 volume in individuals at ultra-high-risk for psychosis who do not remit: findings from the longitudinal youth at risk study. *Neuropsychopharmacology* 2017;**42**(6).

[24] Kakeda S, Watanabe K, Katsuki A*, et al*. Relationship between interleukin (IL)-6 and brain morphology in drug-naïve, first-episode major depressive disorder using surface-based morphometry. *Scientific reports* 2018;**8**(1).

[25] Kang DW, Lim HK, Joo S*, et al*. The association between hippocampal subfield volumes and education in cognitively normal older adults and amnestic mild cognitive impairment patients. *Neuropsychiatric disease and treatment* 2018;**14**.

[26] Kreilkamp B, Weber B, Elkommos SB*, et al*. Hippocampal subfield segmentation in temporal lobe epilepsy: Relation to outcomes. *Acta Neurol Scand* 2018;**137**(6).

[27] Li Y, Zhou W, Dong H*, et al*. Lower Fractional Anisotropy in the Gray Matter of Amygdala-Hippocampus-Nucleus Accumbens Circuit in Methamphetamine Users: an In Vivo Diffusion Tensor Imaging Study. *Neurotoxicity research* 2018;**33**(4).

[28] Li W, Li K, Guan P*, et al*. Volume alteration of hippocampal subfields in first-episode antipsychotic-naïve schizophrenia patients before and after acute antipsychotic treatment. *NeuroImage: Clinical* 2018;**20**.

[29] Long J, Feng Y, Liao H*, et al*. Motor Sequence Learning Is Associated With Hippocampal Subfield Volume in Humans With Medial Temporal Lobe Epilepsy. *Frontiers in human neuroscience* 2018;**12**.

[30] Lv X, He H, Yang Y*, et al*. Radiation-induced hippocampal atrophy in patients with nasopharyngeal carcinoma early after radiotherapy: a longitudinal MR-based hippocampal subfield analysis. *Brain imaging and behavior* 2018;.

[31] Mak E, Donaghy PC, McKiernan E*, et al*. Beta-amyloid deposition maps onto hippocampal and subiculum atrophy in dementia with Lewy bodies. *Neurobiol Aging* 2018;.

[32] Mak E, Gabel S, Su L*, et al*. Multi-modal MRI investigation of volumetric and microstructural changes in the hippocampus and its subfields in mild cognitive impairment, Alzheimer's disease, and dementia with Lewy bodies. *International psychogeriatrics* 2017;**29**(4).

[33] Marečková K, Mareček R, Bencurova P*, et al*. Perinatal stress and human hippocampal volume: Findings from typically developing young adults. *Scientific reports* 2018;**8**(1).

[34] Mole TB, Mak E, Chien Y*, et al*. Dissociated accumbens and hippocampal structural abnormalities across obesity and alcohol dependence. *International Journal of Neuropsychopharmacology* 2016;**19**(9).

[35] Mosher VA, Swain MG, Pang JX*, et al*. Magnetic resonance imaging evidence of hippocampal structural changes in patients with primary biliary cholangitis. *Clinical and translational gastroenterology* 2018;**9**(7).

[36] Müller-Ehrenberg L, Riphagen JM, Verhey FR*, et al*. Alzheimer’s Disease Biomarkers Have Distinct Associations with Specific Hippocampal Subfield Volumes. *J Alzheimer's Dis* 2018;(Preprint).

[37] Ørbo MC, Vangberg TR, Tande PM*, et al*. Memory performance, global cerebral volumes and hippocampal subfield volumes in long-term survivors of Out-of-Hospital Cardiac Arrest. *Resuscitation* 2018;**126**.

[38] Pešlová E, Mareček R, Shaw DJ*, et al*. Hippocampal involvement in nonpathological déjà vu: Subfield vulnerability rather than temporal lobe epilepsy equivalent. *Brain and behavior* 2018;.

[39] Roddy DW, Farrell C, Doolin K*, et al*. The Hippocampus in Depression: More than the sum of its parts? Advanced Hippocampal Substructure Segmentation in Depression. *Biol Psychiatry* 2018;.

[40] Rutland JW, Feldman RE, Delman BN*, et al*. Subfield-specific tractography of the hippocampus in epilepsy patients at 7 Tesla. *Seizure* 2018;**62**.

[41] Sarica A, Vasta R, Novellino F*, et al*. MRI Asymmetry Index of Hippocampal Subfields Increases Through the Continuum From the Mild Cognitive Impairment to the Alzheimer's Disease. *Frontiers in neuroscience* 2018;**12**.

[42] Shim G, Choi K, Kim D*, et al*. Predicting neurocognitive function with hippocampal volumes and DTI metrics in patients with Alzheimer's dementia and mild cognitive impairment. *Brain and behavior* 2017;**7**(9).

[43] Smagula SF, Karim HT, Rangarajan A*, et al*. Association of hippocampal substructure resting-state functional connectivity with memory performance in older adults. *The American Journal of Geriatric Psychiatry* 2018;**26**(6).

[44] Sone D, Sato N, Maikusa N*, et al*. Automated subfield volumetric analysis of hippocampus in temporal lobe epilepsy using high-resolution T2-weighed MR imaging. *NeuroImage: Clinical* 2016;**12**.

[45] Tamnes CK, Bos MG, van de Kamp, Ferdi C*, et al*. Longitudinal development of hippocampal subregions from childhood to adulthood. *Developmental cognitive neuroscience* 2018;**30**.

[46] Tannous J, Amaral-Silva H, Cao B*, et al*. Hippocampal subfield volumes in children and adolescents with mood disorders. *J Psychiatr Res* 2018;**101**.

[47] Teicher MH, Anderson CM, Ohashi K*, et al*. Differential effects of childhood neglect and abuse during sensitive exposure periods on male and female hippocampus. *Neuroimage* 2018;**169**.

[48] Uribe C, Segura B, Baggio HC*, et al*. Differential progression of regional hippocampal atrophy in aging and Parkinson’s disease. *Frontiers in Aging Neuroscience* 2018;**10**.

[49] van der Meer D, Rokicki J, Kaufmann T*, et al*. Brain scans from 21297 individuals reveal the genetic architecture of hippocampal subfield volumes. *bioRxiv* 2018;.

[50] Watanabe R, Kakeda S, Watanabe K*, et al*. Relationship between the hippocampal shape abnormality and serum cortisol levels in first‐episode and drug‐naïve major depressive disorder patients. *Depress Anxiety* 2017;**34**(5).

[51] Whelan CD, Hibar DP, van Velzen LS*, et al*. Heritability and reliability of automatically segmented human hippocampal formation subregions. *Neuroimage* 2016;**128**.

[52] Worker A, Dima D, Combes A*, et al*. Test–retest reliability and longitudinal analysis of automated hippocampal subregion volumes in healthy ageing and A lzheimer's disease populations. *Hum Brain Mapp* 2018;**39**(4).

[53] Zhu B, Chen C, Dang X*, et al*. Hippocampal subfields' volumes are more relevant to fluid intelligence than verbal working memory. *Intelligence* 2017;**61**.

[54] Han K, Won E, Kang J*, et al*. TESC gene-regulating genetic variant (rs7294919) affects hippocampal subfield volumes and parahippocampal cingulum white matter integrity in major depressive disorder. *J Psychiatr Res* 2017;**93**.

[55] Myrvang AD, Vangberg TR, Stedal K*, et al*. Hippocampal Subfields in Adolescent Anorexia Nervosa. *Psychiatry Research: Neuroimaging* 2018;. <http://www.sciencedirect.com/science/article/pii/S0925492718301550>. doi://doi.org/10.1016/j.pscychresns.2018.10.007.

[56] McHugo M, Talati P, Woodward ND*, et al*. Regionally specific volume deficits along the hippocampal long axis in early and chronic psychosis. *NeuroImage: Clinical* 2018;**20**. <http://www.sciencedirect.com/science/article/pii/S2213158218303310>. doi://doi.org/10.1016/j.nicl.2018.10.021.

[57] Lenka A, Ingalhalikar M, Shah A*, et al*. Hippocampal subfield atrophy in patients with Parkinson’s disease and psychosis. *J Neural Transm* 2018;.

[58] Buchy L, Barbato M, MacMaster FP*, et al*. Cognitive insight is associated with cortical thickness in first-episode psychosis. *Schizophr Res* 2016;**172**(1-3).

[59] Stamenova V, Nicola R, Aharon-Peretz J*, et al*. Long-term effects of brief hypoxia due to cardiac arrest: Hippocampal reductions and memory deficits. *Resuscitation* 2018;**126**.

[60] van Velzen LS, Wijdeveld M, Black CN*, et al*. Oxidative stress and brain morphology in individuals with depression, anxiety and healthy controls. *Prog Neuro-Psychopharmacol Biol Psychiatry* 2017;**76**.

[61] Chen X, Fu J, Luo Q*, et al*. Altered volume and microstructural integrity of hippocampus in NMOSD. *Multiple sclerosis and related disorders* 2019;**28**.

[62] Hanert A, Rave J, Granert O*, et al*. Hippocampal dentate gyrus atrophy predicts pattern separation impairment in patients with LGI1 encephalitis. *Neuroscience* 2019;.

[63] Peixoto-Santos JE, de Carvalho, Luciana Estefani Drumond, Kandratavicius L*, et al*. Manual Hippocampal Subfield Segmentation Using High-Field MRI: Impact of Different Subfields in Hippocampal Volume Loss of Temporal Lobe Epilepsy Patients. *Frontiers in neurology* 2018;**9**.

[64] Parker TD, Slattery CF, Yong KX*, et al*. Differences in hippocampal subfield volume are seen in phenotypic variants of early onset Alzheimer's disease. *NeuroImage: Clinical* 2018;.

[65] Elman JA, Panizzon MS, Gillespie NA*, et al*. Genetic architecture of hippocampal subfields on standard resolution MRI: How the parts relate to the whole. *Hum Brain Mapp* 2018;.

[66] Ahmed-Leitao F, Rosenstein D, Marx M*, et al*. Posttraumatic stress disorder, social anxiety disorder and childhood trauma: differences in hippocampal subfield volume. *Psychiatry Research: Neuroimaging* 2018;.

[67] Boccardi V, Westman E, Pelini L*, et al*. Differential associations of IL-4 with hippocampal subfields in Mild Cognitive Impairment and Alzheimer’s disease. *Frontiers in Aging Neuroscience* 2018;**10**.

[68] Low A, Foo H, Yong TT*, et al*. Hippocampal subfield atrophy of CA1 and subicular structures predict progression to dementia in idiopathic Parkinson’s disease. *J Neurol Neurosurg Psychiatr* 2019;.

[69] Marizzoni M, Ferrari C, Jovicich J*, et al*. Predicting and Tracking Short Term Disease Progression in Amnestic Mild Cognitive Impairment Patients with Prodromal Alzheimer’s Disease: Structural Brain Biomarkers. *J Alzheimer's Dis* 2018;(Preprint).

[70] Peter J, Sandkamp R, Minkova L*, et al*. Real-world navigation in amnestic mild cognitive impairment: The relation to visuospatial memory and volume of hippocampal subregions. *Neuropsychologia* 2018;**109**.

[71] Zuppichini MD, Sandry J. Pilot investigation of the relationship between hippocampal volume and pattern separation deficits in multiple sclerosis. *Multiple sclerosis and related disorders* 2018;**26**.

[72] Han K, Kim A, Kang W*, et al*. Hippocampal subfield volumes in major depressive disorder and bipolar disorder. *European Psychiatry* 2019;**57**.

[73] Malhi GS, Das P, Outhred T*, et al*. The effects of childhood trauma on adolescent hippocampal subfields. *Australian & New Zealand Journal of Psychiatry* 2019;.

[74] Aanes S, Bjuland KJ, Sripada K*, et al*. Reduced hippocampal subfield volumes and memory function in school-aged children born preterm with very low birthweight (VLBW). *NeuroImage: Clinical* 2019;**23**.

[75] Alnæs D, Kaufmann T, van der Meer D*, et al*. Brain heterogeneity in schizophrenia and its association with polygenic risk. *JAMA psychiatry* 2019;.

[76] Bocchetta M, Iglesias JE, Russell LL*, et al*. Segmentation of medial temporal subregions reveals early right-sided involvement in semantic variant PPA. *Alzheimer's research & therapy* 2019;**11**(1).

[77] Brown S, Rutland JW, Verma G*, et al*. Structural MRI at 7T reveals amygdala nuclei and hippocampal subfield volumetric association with Major Depressive Disorder symptom severity. *Scientific reports* 2019;**9**(1).

[78] Carey D, Nolan H, Kenny RA*, et al*. Dissociable age and memory relationships with hippocampal subfield volumes in vivo: Data from the Irish Longitudinal study on Ageing (tILDA). *Scientific reports* 2019;**9**.

[79] Hedderich DM, Reess TJ, Thaler M*, et al*. Hippocampus subfield volumetry after microsurgical or endovascular treatment of intracranial aneurysms—an explorative study. *European radiology experimental* 2019;**3**(1).

[80] Jiang L, Cao X, Jiang J*, et al*. Atrophy of hippocampal subfield CA2/3 in healthy elderly men is related to educational attainment. *Neurobiol Aging* 2019;**80**.

[81] Kraus C, Seiger R, Pfabigan DM*, et al*. Hippocampal Subfields in Acute and Remitted Depression—an Ultra-High Field Magnetic Resonance Imaging Study. *International Journal of Neuropsychopharmacology* 2019;**22**(8).

[82] Merz EC, Desai PM, Maskus EA*, et al*. Socioeconomic Disparities in Chronic Physiologic Stress Are Associated with Brain Structure in Children. *Biol Psychiatry* 2019;.

[83] Mikolas P, Tozzi L, Doolin K*, et al*. Effects of early life adversity and FKBP5 genotype on hippocampal subfields volume in major depression. *J Affect Disord* 2019;**252**.

[84] Otsuka Y, Kakeda S, Sugimoto K*, et al*. COMT polymorphism regulates the hippocampal subfield volumes in first-episode, drug-naive patients with major depressive disorder. *Neuropsychiatric Disease and Treatment* 2019;**15**.

[85] Prasad S, Shah A, Bhalsing KS*, et al*. Abnormal hippocampal subfields are associated with cognitive impairment in essential tremor. *J Neural Transm* 2019;**126**(5).

[86] Rahayel S, Bocti C, Sévigny Dupont P*, et al*. Subcortical amyloid load is associated with shape and volume in cognitively normal individuals. *Hum Brain Mapp* 2019;.

[87] Rutland JW, Brown S, Verma G*, et al*. Hippocampal subfield-specific connectivity findings in major depressive disorder: A 7 Tesla diffusion MRI study. *J Psychiatr Res* 2019;**111**.

[88] Vaculik M, Noorani A, Hung PS*, et al*. Selective hippocampal subfield volume reductions in classic trigeminal neuralgia. *NeuroImage: Clinical* 2019;.

[89] Wang N, Zhang L, Yang H*, et al*. Do multiple system atrophy and Parkinson’s disease show distinct patterns of volumetric alterations across hippocampal subfields? An exploratory study. *Eur Radiol* 2019;.

[90] Zahr NM, Pohl KM, Saranathan M*, et al*. Hippocampal subfield CA2 3 exhibits accelerated aging in Alcohol Use Disorder: A preliminary study. *NeuroImage: Clinical* 2019;**22**.

[91] Zhao W, Wang X, Yin C*, et al*. Trajectories of The Hippocampal Subfields Atrophy in the Alzheimer’s Disease: A Structural Imaging Study. *Frontiers in neuroinformatics* 2019;**13**.

[92] Zhang L, Hu X, Lu L*, et al*. Abnormalities of hippocampal shape and subfield volumes in medication‐free patients with obsessive–compulsive disorder. *Hum Brain Mapp* 2019;.

[93] Zheng F, Cui D, Zhang L*, et al*. The volume of hippocampal subfields in relation to decline of memory recall across the adult lifespan. *Frontiers in aging neuroscience* 2018;**10**.

[94] Alkan E, Davies G, Greenwood K*, et al*. Brain Structural Correlates of Metacognition in First-Episode Psychosis. *Schizophr Bull* 2019;.

[95] Alves JM, Luo S, Chow T*, et al*. Sex differences in the association between prenatal exposure to maternal obesity and hippocampal volume in children. *Brain and behavior* 2020;**10**(2).

[96] Broadhouse KM, Singh MF, Suo C*, et al*. Hippocampal plasticity underpins long-term cognitive gains from resistance exercise in MCI. *NeuroImage: Clinical* 2020;.

[97] Broadhouse KM, Mowszowski L, Duffy S*, et al*. Memory Performance Correlates of Hippocampal Subfield Volume in Mild Cognitive Impairment Subtype. *Frontiers in Behavioral Neuroscience* 2019;**13**.

[98] Christidi F, Karavasilis E, Rentzos M*, et al*. Hippocampal pathology in amyotrophic lateral sclerosis: selective vulnerability of subfields and their associated projections. *Neurobiol Aging* 2019;**84**.

[99] Christidi F, Karavasilis E, Rentzos M*, et al*. Neuroimaging data indicate divergent mesial temporal lobe profiles in amyotrophic lateral sclerosis, Alzheimer's disease and healthy aging. *Data in brief* 2020;**28**.

[100] Garimella A, Rajguru S, Singla U*, et al*. Marijuana and the hippocampus: A longitudinal study on the effects of marijuana on hippocampal subfields. *Prog Neuro-Psychopharmacol Biol Psychiatry* 2020;.

[101] Gryglewski G, Baldinger-Melich P, Seiger R*, et al*. Structural changes in amygdala nuclei, hippocampal subfields and cortical thickness following electroconvulsive therapy in treatment-resistant depression: longitudinal analysis. *The British Journal of Psychiatry* 2019;**214**(3).

[102] Herting MM, Azad A, Kim R*, et al*. Brain Differences in the Prefrontal Cortex, Amygdala, and Hippocampus in Youth with Congenital Adrenal Hyperplasia. *The Journal of Clinical Endocrinology & Metabolism* 2020;**105**(4).

[103] Nogovitsyn N, Souza R, Muller M*, et al*. Aberrant limbic brain structures in young individuals at risk for mental illness. *Psychiatry Clin Neurosci* 2020;.

[104] Parker TD, Cash DM, Lane CA*, et al*. Hippocampal subfield volumes and pre-clinical Alzheimer’s disease in 408 cognitively normal adults born in 1946. *PloS one* 2019;**14**(10).

[105] du Plessis S, Scheffler F, Luckhoff H*, et al*. Childhood trauma and hippocampal subfield volumes in first-episode schizophrenia and healthy controls. *Schizophr Res* 2020;**215**.

[106] Sala-Padro J, Miró J, Rodriguez-Fornells A*, et al*. Hippocampal microstructural architecture and surgical outcome: Hippocampal diffusivity could predict seizure relapse. *Seizure* 2020;**76**.

[107] Tannous J, Godlewska BR, Tirumalaraju V*, et al*. Stress, inflammation and hippocampal subfields in depression: A 7 Tesla MRI Study. *Translational Psychiatry* 2020;**10**(1).

[108] Tesli N, van der Meer D, Rokicki J*, et al*. Hippocampal subfield and amygdala nuclei volumes in schizophrenia patients with a history of violence. *Eur Arch Psychiatry Clin Neurosci* 2020;.

[109] Wang J, Wu S, Sun Y*, et al*. Interaction of COMT and KIBRA modulates the association between hippocampal structure and episodic memory performance in healthy young adults. *Behav Brain Res* 2020;**384**.

[110] Xu R, Hu X, Jiang X*, et al*. Longitudinal volume changes of hippocampal subfields and cognitive decline in Parkinson's disease. *QUANTITATIVE IMAGING IN MEDICINE AND SURGERY* 2020;**10**(1).

[111] Zhang L, Mak E, Reilhac A*, et al*. Longitudinal trajectory of Amyloid‐related hippocampal subfield atrophy in nondemented elderly. *Hum Brain Mapp* ;.

[112] Zhou D, Liu S, Zhou X*, et al*. 7 T MRI reveals hippocampal structural abnormalities associated with memory intrusions in childhood-onset schizophrenia. *Schizophr Res* 2018;**202**.

[113] Zheng F, Li C, Zhang D*, et al*. Study on the sub-regions volume of hippocampus and amygdala in schizophrenia. *Quantitative imaging in medicine and surgery* 2019;**9**(6).

[114] Dounavi M, Mak E, Wells K*, et al*. Volumetric alterations in the hippocampal subfields of subjects at increased risk of dementia. *Neurobiol Aging* 2020;.

[115] Al‐Amin M, Bradford D, Sullivan RK*, et al*. Vitamin D deficiency is associated with reduced hippocampal volume and disrupted structural connectivity in patients with mild cognitive impairment. *Hum Brain Mapp* 2019;**40**(2).

[116] Costa BS, Santos MCV, Rosa DV*, et al*. Automated evaluation of hippocampal subfields volumes in mesial temporal lobe epilepsy and its relationship to the surgical outcome. *Epilepsy Res* 2019;**154**.

[117] Ernst L, David B, Gaubatz J*, et al*. Volumetry of Mesiotemporal Structures Reflects Serostatus in Patients with Limbic Encephalitis. *Am J Neuroradiol* 2019;**40**(12).

[118] Finegan E, Shing SLH, Chipika RH*, et al*. Widespread subcortical grey matter degeneration in primary lateral sclerosis: a multimodal imaging study with genetic profiling. *NeuroImage: Clinical* 2019;**24**.

[119] Gicas KM, Thornton AE, Waclawik K*, et al*. Volumes of the hippocampal formation differentiate component processes of memory in a community sample of homeless and marginally housed persons. *Archives of Clinical Neuropsychology* 2018;**34**(4).

[120] Maksimovskiy AL, Oot EN, Seraikas AM*, et al*. Morphometric Biomarkers of Adolescents With Familial Risk for Alcohol Use Disorder. *Alcoholism: Clinical and Experimental Research* 2019;**43**(11).

[121] Shim J, Kim Y, Kim S*, et al*. Volumetric reductions of subcortical structures and their localizations in alcohol dependent patients. *Frontiers in neurology* 2019;**10**.

[122] Smeeth DM, Dima D, Jones L*, et al*. Polygenic risk for circulating reproductive hormone levels and their influence on hippocampal volume and depression susceptibility. *Psychoneuroendocrinology* 2019;**106**.

[123] Zheng F, Liu Y, Yuan Z*, et al*. Age‐related changes in cortical and subcortical structures of healthy adult brains: A surface‐based morphometry study. *Journal of Magnetic Resonance Imaging* 2019;**49**(1).

[124] Axelrud LK, Santoro ML, Pine DS*, et al*. Polygenic Risk Score for Alzheimer’s Disease: Implications for Memory Performance and Hippocampal Volumes in Early Life. *Am J Psychiatry* 2018;**175**(6).

[125] Decker AL, Szulc KU, Bouffet E*, et al*. Smaller hippocampal subfield volumes predict verbal associative memory in pediatric brain tumor survivors. *Hippocampus* 2017;**27**(11).

[126] Kälin AM, Park M, Chakravarty MM*, et al*. Subcortical Shape Changes, Hippocampal Atrophy and Cortical Thinning in Future Alzheimer's Disease Patients. *Frontiers in aging neuroscience* 2017;**9**.

[127] Leh SE, Schroeder C, Chen J*, et al*. Microstructural integrity of hippocampal subregions is impaired after mild traumatic brain injury. *J Neurotrauma* 2017;**34**(7).

[128] Romero JE, Coupe P, Manjon JV. HIPS: A new hippocampus subfield segmentation method. *Neuroimage* 2017;**163**. doi:S1053-8119(17)30797-8 [pii].

[129] Schroeder C, Park MTM, Germann J*, et al*. Hippocampal shape alterations are associated with regional Aβ load in cognitively normal elderly individuals. *Eur J Neurosci* 2017;**45**(10).

[130] Sussman D, Leung RC, Vogan VM*, et al*. The autism puzzle: Diffuse but not pervasive neuroanatomical abnormalities in children with ASD. *NeuroImage: Clinical* 2015;**8**.

[131] Treadway MT, Waskom ML, Dillon DG*, et al*. Illness progression, recent stress, and morphometry of hippocampal subfields and medial prefrontal cortex in major depression. *Biol Psychiatry* 2015;**77**(3).

[132] Wannan CM, Cropley VL, Chakravarty MM*, et al*. Hippocampal subfields and visuospatial associative memory across stages of schizophrenia-spectrum disorder. *Psychol Med* 2018;.

[133] Eguchi Y, Noda Y, Nakajima S*, et al*. Subiculum volumes associated with memory function in the oldest‐old individuals aged 95 years and older. *Geriatrics & gerontology international* 2019;**19**(4).

[134] Chau CM, Ranger M, Bichin M*, et al*. Hippocampus, Amygdala, and Thalamus Volumes in Very Preterm Children at 8 Years: Neonatal Pain and Genetic Variation. *Frontiers in behavioral neuroscience* 2019;**13**.

[135] Takamiya A, Plitman E, Chung JK*, et al*. Acute and long-term effects of electroconvulsive therapy on human dentate gyrus. *Neuropsychopharmacology* 2019;**44**(10).

[136] Aas M, Haukvik UK, Djurovic S*, et al*. Interplay between childhood trauma and BDNF val66met variants on blood BDNF mRNA levels and on hippocampus subfields volumes in schizophrenia spectrum and bipolar disorders. *J Psychiatr Res* 2014;**59**.

[137] Abbott CC, Jones T, Lemke NT*, et al*. Hippocampal structural and functional changes associated with electroconvulsive therapy response. *Translational psychiatry* 2014;**4**(11).

[138] Batalla A, Lorenzetti V, Chye Y*, et al*. The Influence of DAT1, COMT, and BDNF Genetic Polymorphisms on Total and Subregional Hippocampal Volumes in Early Onset Heavy Cannabis Users. *Cannabis and cannabinoid research* 2018;**3**(1).

[139] Kim JB, Suh S, Kim JH. Volumetric and shape analysis of hippocampal subfields in unilateral mesial temporal lobe epilepsy with hippocampal atrophy. *Epilepsy Res* 2015;**117**.

[140] Boen E, Westlye LT, Elvsashagen T*, et al*. Smaller stress-sensitive hippocampal subfields in women with borderline personality disorder without posttraumatic stress disorder. *J Psychiatry Neurosci* 2014;**39**(2). doi:10.1503/jpn.130070 [pii].

[141] Booij L, Szyf M, Carballedo A*, et al*. DNA methylation of the serotonin transporter gene in peripheral cells and stress-related changes in hippocampal volume: a study in depressed patients and healthy controls. *PloS one* 2015;**10**(3).

[142] Brickman AM, Khan UA, Provenzano FA*, et al*. Enhancing dentate gyrus function with dietary flavanols improves cognition in older adults. *Nat Neurosci* 2014;**17**(12).

[143] Burkert NT, Koschutnig K, Ebner F*, et al*. Structural hippocampal alterations, perceived stress, and coping deficiencies in patients with anorexia nervosa. *Int J Eat Disord* 2015;**48**(6).

[144] Caldairou B, Bernhardt BC, Kulaga-Yoskovitz J*, et al*. A surface patch-based segmentation method for hippocampal subfields. 2016;.

[145] Carlesimo GA, Piras F, Orfei MD*, et al*. Atrophy of presubiculum and subiculum is the earliest hippocampal anatomical marker of Alzheimer's disease. *Alzheimer's & Dementia: Diagnosis, Assessment & Disease Monitoring* 2015;**1**(1).

[146] Cha J, Greenberg T, Song I*, et al*. Abnormal hippocampal structure and function in clinical anxiety and comorbid depression. *Hippocampus* 2016;**26**(5).

[147] Chalavi S, Vissia EM, Giesen ME*, et al*. Abnormal hippocampal morphology in dissociative identity disorder and post‐traumatic stress disorder correlates with childhood trauma and dissociative symptoms. *Hum Brain Mapp* 2015;**36**(5).

[148] Chen L, Luo T, Lv F*, et al*. Relationship between hippocampal subfield volumes and memory deficits in patients with thalamus infarction. *Eur Arch Psychiatry Clin Neurosci* 2016;**266**(6).

[149] Chye Y, Suo C, Yücel M*, et al*. Cannabis-related hippocampal volumetric abnormalities specific to subregions in dependent users. *Psychopharmacology (Berl )* 2017;**234**(14).

[150] Dahmen B, Puetz VB, Scharke W*, et al*. Effects of early-life adversity on hippocampal structures and associated HPA axis functions. *Dev Neurosci* 2018;**40**(1).

[151] de Flores R, La Joie R, Landeau B*, et al*. Effects of age and Alzheimer's disease on hippocampal subfields: comparison between manual and FreeSurfer volumetry. *Hum Brain Mapp* 2015;**36**(2). doi:10.1002/hbm.22640 [doi].

[152] Pizzi SD, Franciotti R, Bubbico G*, et al*. Atrophy of hippocampal subfields and adjacent extrahippocampal structures in dementia with Lewy bodies and Alzheimer's disease. *Neurobiol Aging* 2016;**40**.

[153] Donnelly-Kehoe PA, Pascariello GO, Gómez JC*, et al*. Looking for Alzheimer's Disease morphometric signatures using machine learning techniques. *J Neurosci Methods* 2018;**302**.

[154] Durazzo TC, Meyerhoff DJ, Nixon SJ. Interactive effects of chronic cigarette smoking and age on hippocampal volumes. *Drug Alcohol Depend* 2013;**133**(2).

[155] Elvsåshagen T, Westlye LT, Bøen E*, et al*. Evidence for reduced dentate gyrus and fimbria volume in bipolar II disorder. *Bipolar Disord* 2013;**15**(2).

[156] Elvsåshagen T, Zuzarte P, Westlye LT*, et al*. Dentate gyrus− cornu ammonis (CA) 4 volume is decreased and associated with depressive episodes and lipid peroxidation in bipolar II disorder: Longitudinal and cross‐sectional analyses. *Bipolar Disord* 2016;**18**(8).

[157] Engvig A, Fjell AM, Westlye LT*, et al*. Hippocampal subfield volumes correlate with memory training benefit in subjective memory impairment. *Neuroimage* 2012;**61**(1).

[158] Ezzati A, Zimmerman ME, Katz MJ*, et al*. Hippocampal subfields differentially correlate with chronic pain in older adults. *Brain Res* 2014;**1573**.

[159] Ezzati A, Katz MJ, Lipton ML*, et al*. The association of brain structure with gait velocity in older adults: a quantitative volumetric analysis of brain MRI. *Neuroradiology* 2015;**57**(8).

[160] Feng X, Hamberger MJ, Sigmon HC*, et al*. Temporal lobe epilepsy lateralization using retrospective cerebral blood volume MRI. *NeuroImage: Clinical* 2018;.

[161] Finke C, Prüss H, Heine J*, et al*. Evaluation of cognitive deficits and structural hippocampal damage in encephalitis with leucine-rich, glioma-inactivated 1 antibodies. *JAMA neurology* 2017;**74**(1).

[162] Finke C, Kopp UA, Pajkert A*, et al*. Structural hippocampal damage following anti-N-methyl-D-aspartate receptor encephalitis. *Biol Psychiatry* 2016;**79**(9).

[163] Fleischman DA, Arfanakis K, Leurgans S*, et al*. Neopterin is associated with hippocampal subfield volumes and cognition in HIV. *Neurology-Neuroimmunology Neuroinflammation* 2018;**5**(4).

[164] Foo H, Mak E, Yong TT*, et al*. Progression of subcortical atrophy in mild Parkinson's disease and its impact on cognition. *European journal of neurology* 2017;**24**(2).

[165] Frodl T, Skokauskas N, Frey E*, et al*. BDNF V al66 M et genotype interacts with childhood adversity and influences the formation of hippocampal subfields. *Hum Brain Mapp* 2014;**35**(12).

[166] Frodl T, Carballedo A, Frey E*, et al*. Expression of glucocorticoid inducible genes is associated with reductions in cornu ammonis and dentate gyrus volumes in patients with major depressive disorder. *Dev Psychopathol* 2014;**26**(4pt2).

[167] Germeyan SC, Kalikhman D, Jones L*, et al*. Automated versus manual hippocampal segmentation in preoperative and postoperative patients with epilepsy. *Epilepsia* 2014;**55**(9).

[168] Giakoumatos CI, Nanda P, Mathew IT*, et al*. Effects of lithium on cortical thickness and hippocampal subfield volumes in psychotic bipolar disorder. *J Psychiatr Res* 2015;**61**.

[169] Han K, Won E, Sim Y*, et al*. Hippocampal subfield analysis in medication-naive female patients with major depressive disorder. *J Affect Disord* 2016;**194**.

[170] Harel EV, Tennyson RL, Fava M*, et al*. Linking major depression and the neural substrates of associative processing. *Cognitive, Affective, & Behavioral Neuroscience* 2016;**16**(6).

[171] Hartberg CB, Jørgensen KN, Haukvik UK*, et al*. Lithium treatment and hippocampal subfields and amygdala volumes in bipolar disorder. *Bipolar Disord* 2015;**17**(5).

[172] Haukvik UK, Westlye LT, Mørch-Johnsen L*, et al*. In vivo hippocampal subfield volumes in schizophrenia and bipolar disorder. *Biol Psychiatry* 2015;**77**(6).

[173] Hayes JP, Hayes S, Miller DR*, et al*. Automated measurement of hippocampal subfields in PTSD: evidence for smaller dentate gyrus volume. *J Psychiatr Res* 2017;**95**.

[174] Hirjak D, Wolf RC, Remmele B*, et al*. Hippocampal formation alterations differently contribute to autobiographic memory deficits in mild cognitive impairment and Alzheimer's disease. *Hippocampus* 2017;**27**(6).

[175] Hirjak D, Sambataro F, Remmele B*, et al*. The relevance of hippocampal subfield integrity and clock drawing test performance for the diagnosis of Alzheimer’s disease and mild cognitive impairment. *The World Journal of Biological Psychiatry* 2017;.

[176] Hoseth EZ, Westlye LT, Hope S*, et al*. Association between cytokine levels, verbal memory and hippocampus volume in psychotic disorders and healthy controls. *Acta Psychiatr Scand* 2016;**133**(1).

[177] Hsu PJ, Shou H, Benzinger T*, et al*. Amyloid burden in cognitively normal elderly is associated with preferential hippocampal subfield volume loss. *J Alzheimer's Dis* 2015;**45**(1).

[178] Hýža M, Kuhn M, Češková E*, et al*. Hippocampal volume in first-episode schizophrenia and longitudinal course of the illness. *The World Journal of Biological Psychiatry* 2016;**17**(6).

[179] Hu X, Zhang L, Hu X*, et al*. Abnormal Hippocampal Subfields May Be Potential Predictors of Worse Early Response to Antidepressant Treatment in Drug‐Naïve Patients With Major Depressive Disorder. *Journal of Magnetic Resonance Imaging* 2018;.

[180] Inlow M, Cong S, Risacher SL*, et al*. A New Statistical Image Analysis Approach and Its Application to Hippocampal Morphometry. 2016;.

[181] Kawano M, Sawada K, Shimodera S*, et al*. Hippocampal subfield volumes in first episode and chronic schizophrenia. *PLoS One* 2015;**10**(2).

[182] Khan W, Westman E, Jones N*, et al*. Automated hippocampal subfield measures as predictors of conversion from mild cognitive impairment to Alzheimer’s disease in two independent cohorts. *Brain Topogr* 2015;**28**(5).

[183] Köbe T, Witte AV, Schnelle A*, et al*. Vitamin B-12 concentration, memory performance, and hippocampal structure in patients with mild cognitive impairment, 2. *Am J Clin Nutr* 2016;**103**(4).

[184] Koch K, Reess TJ, Rus OG*, et al*. Extensive learning is associated with gray matter changes in the right hippocampus. *Neuroimage* 2016;**125**.

[185] Krogsrud SK, Tamnes CK, Fjell AM*, et al*. Development of hippocampal subfield volumes from 4 to 22 years. *Hum Brain Mapp* 2014;**35**(11).

[186] Kuhn S, Musso F, Mobascher A*, et al*. Hippocampal subfields predict positive symptoms in schizophrenia: first evidence from brain morphometry. *Transl Psychiatry* 2012;**2**(2158-3188; 2158-3188).

[187] Kühn S, Charlet K, Schubert F*, et al*. Plasticity of hippocampal subfield volume cornu ammonis 2 3 over the course of withdrawal in patients with alcohol dependence. *JAMA psychiatry* 2014;**71**(7).

[188] Lee J, Im S, Lee S*, et al*. Volume of hippocampal subfields in patients with alcohol dependence. *Psychiatry Research: Neuroimaging* 2016;**258**.

[189] Lee SW, Yoo JH, Kim KW*, et al*. Hippocampal Subfields Volume Reduction in High Schoolers with Previous Verbal Abuse Experiences. *Clinical Psychopharmacology and Neuroscience* 2018;**16**(1).

[190] Li YD, Dong HB, Xie GM*, et al*. Discriminative Analysis of Mild Alzheimer's Disease and Normal Aging Using Volume of Hippocampal Subfields and Hippocampal Mean Diffusivity: An In Vivo Magnetic Resonance Imaging Study. *Am J Alzheimers Dis Other Demen* 2013;**28**(6). doi:10.1177/1533317513494452.

[191] Eliassen CF, Selnes P, Almdahl IS*, et al*. Hippocampal subfield atrophy in multi-domain but not amnestic mild cognitive impairment. *Dement Geriatr Cogn Disord* 2015;**40**(1-2).

[192] Francis AN, Seidman LJ, Tandon N*, et al*. Reduced subicular subdivisions of the hippocampal formation and verbal declarative memory impairments in young relatives at risk for schizophrenia. *Schizophr Res* 2013;**151**(1-3).

[193] Li X, Li D, Li Q*, et al*. Hippocampal subfield volumetry in patients with subcortical vascular mild cognitive impairment. *Scientific reports* 2016;**6**.

[194] Kook LH, Chul HS, Sang JW*, et al*. Automated hippocampal subfields segmentation in late life depression. *J Affect Disord* 2012;(1573-2517; 0165-0327).

[195] Lim HK, Hong SC, Jung WS*, et al*. Automated segmentation of hippocampal subfields in drug-naive patients with Alzheimer disease. *AJNR Am J Neuroradiol* 2013;**34**(4). doi:10.3174/ajnr.A3293 [doi].

[196] Lim HK, Hong SC, Jung WS*, et al*. Automated hippocampal subfield segmentation in amnestic mild cognitive impairments. *Dement Geriatr Cogn Disord* 2012;**33**(5).

[197] Lindberg O, Mårtensson G, Stomrud E*, et al*. Atrophy of the Posterior Subiculum Is Associated with Memory Impairment, Tau-and Aβ Pathology in Non-demented Individuals. *Frontiers in aging neuroscience* 2017;**9**.

[198] Luo Y, Liu Y, Qin Y*, et al*. The atrophy and laterality of the hippocampal subfields in parents with or without posttraumatic stress disorder who lost their only child in China. *Neurological Sciences* 2017;**38**(7).

[199] Mak E, Su L, Williams GB*, et al*. Differential atrophy of hippocampal subfields: a comparative study of dementia with Lewy bodies and Alzheimer disease. *The American Journal of Geriatric Psychiatry* 2016;**24**(2).

[200] Malchow B, Keeser D, Keller K*, et al*. Effects of endurance training on brain structures in chronic schizophrenia patients and healthy controls. *Schizophr Res* 2016;**173**(3).

[201] Marizzoni M, Antelmi L, Bosch B*, et al*. Longitudinal reproducibility of automatically segmented hippocampal subfields: A multisite European 3T study on healthy elderly. *Hum Brain Mapp* 2015;**36**(9). doi:10.1002/hbm.22859 [doi].

[202] Mathew I, Gardin TM, Tandon N*, et al*. Medial temporal lobe structures and hippocampal subfields in psychotic disorders: findings from the Bipolar-Schizophrenia Network on Intermediate Phenotypes (B-SNIP) study. *JAMA psychiatry* 2014;**71**(7).

[203] Meier TB, Savitz J, Singh R*, et al*. Smaller dentate gyrus and CA2 and CA3 volumes are associated with kynurenine metabolites in collegiate football athletes. *J Neurotrauma* 2016;**33**(14).

[204] Na K, Chang HS, Won E*, et al*. Association between glucocorticoid receptor methylation and hippocampal subfields in major depressive disorder. *PloS one* 2014;**9**(1).

[205] Novellino F, Vasta R, Sarica A*, et al*. Relationship between Hippocampal Subfields and Category Cued Recall in AD and PDD: A Multimodal MRI Study. *Neuroscience* 2018;**371**.

[206] Orfei MD, Piras F, Banaj N*, et al*. Unrealistic self-overconfidence in schizophrenia is associated with left presubiculum atrophy and impaired episodic memory. *Cortex* 2017;**86**.

[207] Pagliaccio D, Luby JL, Bogdan R*, et al*. Stress-system genes and life stress predict cortisol levels and amygdala and hippocampal volumes in children. *Neuropsychopharmacology* 2014;**39**(5).

[208] Papiol S, Popovic D, Keeser D*, et al*. Polygenic risk has an impact on the structural plasticity of hippocampal subfields during aerobic exercise combined with cognitive remediation in multi-episode schizophrenia. *Translational psychiatry* 2017;**7**(6).

[209] Pasquini L, Scherr M, Tahmasian M*, et al*. Increased intrinsic activity of medial-temporal lobe subregions is associated with decreased cortical thickness of medial-parietal areas in patients with Alzheimer’s disease dementia. *J Alzheimer's Dis* 2016;**51**(1).

[210] Pereira JB, Valls-Pedret C, Ros E*, et al*. Regional vulnerability of hippocampal subfields to aging measured by structural and diffusion MRI. *Hippocampus* 2014;**24**(4). doi:10.1002/hipo.22234 [doi].

[211] Pereira JB, Junqué C, Bartrés‐Faz D*, et al*. Regional vulnerability of hippocampal subfields and memory deficits in Parkinson's disease. *Hippocampus* 2013;**23**(8).

[212] Rabl U, Meyer BM, Diers K*, et al*. Additive gene–environment effects on hippocampal structure in healthy humans. *Journal of Neuroscience* 2014;**34**(30).

[213] Reiter K, Nielson KA, Durgerian S*, et al*. Five-year longitudinal brain volume change in healthy elders at genetic risk for Alzheimer’s disease. *J Alzheimer's Dis* 2017;**55**(4).

[214] Schoene‐Bake J, Keller SS, Niehusmann P*, et al*. In vivo mapping of hippocampal subfields in mesial temporal lobe epilepsy: relation to histopathology. *Hum Brain Mapp* 2014;**35**(9).

[215] Selnes P, Grambaite R, Rincon M*, et al*. Hippocampal complex atrophy in poststroke and mild cognitive impairment. *Journal of Cerebral Blood Flow & Metabolism* 2015;**35**(11).

[216] Simonetti A, Sani G, Dacquino C*, et al*. Hippocampal subfield volumes in short‐and long‐term lithium‐treated patients with bipolar I disorder. *Bipolar Disord* 2016;**18**(4).

[217] Stav AL, Johansen KK, Auning E*, et al*. Hippocampal subfield atrophy in relation to cerebrospinal fluid biomarkers and cognition in early Parkinson’s disease: a cross-sectional study. *NPJ Parkinson's disease* 2016;**2**.

[218] Szymkowicz SM, McLaren ME, O'shea A*, et al*. Depressive symptoms modify age effects on hippocampal subfields in older adults. *Geriatrics & gerontology international* 2017;**17**(10).

[219] Tamnes CK, Walhovd KB, Engvig A*, et al*. Regional hippocampal volumes and development predict learning and memory. *Dev Neurosci* 2014;**36**(3-4).

[220] Teicher MH, Anderson CM, Polcari A. Childhood maltreatment is associated with reduced volume in the hippocampal subfields CA3, dentate gyrus, and subiculum. *Proc Natl Acad Sci U S A* 2012;**109**(1091-6490; 0027-8424; 9).

[221] Vargas T, Dean DJ, Osborne KJ*, et al*. Hippocampal Subregions Across the Psychosis Spectrum. *Schizophr Bull* 2017;.

[222] Vasta R, Augimeri A, Cerasa A*, et al*. Hippocampal subfield atrophies in converted and not-converted mild cognitive impairments patients by a markov random fields algorithm. *Current Alzheimer Research* 2016;**13**(5).

[223] Wagner G, Herbsleb M, Cruz Fdl*, et al*. Hippocampal structure, metabolism, and inflammatory response after a 6-week intense aerobic exercise in healthy young adults: a controlled trial. *Journal of Cerebral Blood Flow & Metabolism* 2015;**35**(10).

[224] Wang W, Liu Y, Wang H*, et al*. Impacts of CD33 genetic variations on the atrophy rates of hippocampus and parahippocampal gyrus in normal aging and mild cognitive impairment. *Mol Neurobiol* 2017;**54**(2).

[225] Wang X, Yu Y, Zhao W*, et al*. Altered Whole-Brain Structural Covariance of the Hippocampal Subfields in Subcortical Vascular Mild Cognitive Impairment and Amnestic Mild Cognitive Impairment Patients. *Frontiers in neurology* 2018;**9**.

[226] Westeneng H, Verstraete E, Walhout R*, et al*. Subcortical structures in amyotrophic lateral sclerosis. *Neurobiol Aging* 2015;**36**(2).

[227] Whelan CD, Hibar DP, van Velzen LS*, et al*. Heritability and reliability of automatically segmented human hippocampal formation subregions. *Neuroimage* 2015;**128**. doi:S1053-8119(15)01152-0 [pii].

[228] Whittle S, Simmons JG, Hendriksma S*, et al*. Childhood maltreatment, psychopathology, and the development of hippocampal subregions during adolescence. *Brain and behavior* 2017;**7**(2).

[229] Witte AV, Köbe T, Kerti L*, et al*. Impact of KIBRA polymorphism on memory function and the hippocampus in older adults. *Neuropsychopharmacology* 2016;**41**(3).

[230] Witte AV, Köbe T, Graunke A*, et al*. Impact of leptin on memory function and hippocampal structure in mild cognitive impairment. *Hum Brain Mapp* 2016;**37**(12).

[231] Wu Z, Gao Y, Shi F*, et al*. Segmenting hippocampal subfields from 3T MRI with multi-modality images. *Med Image Anal* 2018;**43**. doi:S1361-8415(17)30137-8 [pii].

[232] Zammit AR, Ezzati A, Zimmerman ME*, et al*. Roles of hippocampal subfields in verbal and visual episodic memory. *Behav Brain Res* 2017;**317**.

[233] Zhang Y, Zhang J, Liu C*, et al*. Memory dysfunction in type 2 diabetes mellitus correlates with reduced hippocampal CA1 and subiculum volumes. *Chin Med J* 2015;**128**(4).

[234] Zheng F, Cui D, Zhang L*, et al*. The volume of hippocampal subfields in relation to decline of memory recall across the adult lifespan. *Frontiers in Aging Neuroscience* 2018;**10**.

[235] Zimmerman ME, Ezzati A, Katz MJ*, et al*. Perceived stress is differentially related to hippocampal subfield volumes among older adults. *PloS one* 2016;**11**(5).

[236] Frodl T, Strehl K, Carballedo A*, et al*. Aerobic exercise increases hippocampal subfield volumes in younger adults and prevents volume decline in the elderly. *Brain imaging and behavior* 2019;.

[237] Sánchez AG, Zapata JO. Hippocampal sclerosis: Volumetric evaluation of the substructures of the hippocampus by magnetic resonance imaging. *Radiología (English Edition)* 2018;**60**(5).

[238] Janiri D, Sani G, De Rossi P*, et al*. Hippocampal subfield volumes and childhood trauma in bipolar disorders. *J Affect Disord* 2019;**253**.

[239] Park J, Lee C, Sim Y*, et al*. Automated Subfield Volumetric Analysis of Hippocampus in Patients with Drug-Naïve Nondementia Parkinson’s Disease. *Parkinson’s Disease* 2019;**2019**.

[240] Takaishi M, Asami T, Yoshida H*, et al*. Smaller volume of right hippocampal CA2/3 in patients with panic disorder. *Brain Imaging and Behavior* 2020;.

[241] Izzo J, Andreassen OA, Westlye LT*, et al*. The association between hippocampal subfield volumes in mild cognitive impairment and conversion to Alzheimer’s disease. *Brain Res* 2020;**1728**.

[242] Duan Y, Lin Y, Rosen D*, et al*. Identifying Morphological Patterns of Hippocampal Atrophy in Patients With Mesial Temporal Lobe Epilepsy and Alzheimer Disease. *Frontiers in Neurology* 2020;**11**.

[243] Campabadal A, Segura B, Junque C*, et al*. Cortical gray matter and hippocampal atrophy in idiopathic Rapid Eye Movement sleep behavior disorder. *Frontiers in neurology* 2019;**10**.

[244] Janiri D, Simonetti A, Piras F*, et al*. Predominant polarity and hippocampal subfield volumes in Bipolar disorders. *Bipolar Disord* 2019;.

[245] Liang X, Yin Z, Liu R*, et al*. The Role of MRI Biomarkers and Their Interactions with Cognitive Status and APOE ε4 in Nondemented Elderly Subjects. *Neurodegenerative Diseases* 2018;**18**(5-6).

[246] Tardif CL, Devenyi GA, Amaral RS*, et al*. Regionally specific changes in the hippocampal circuitry accompany progression of cerebrospinal fluid biomarkers in preclinical Alzheimer's disease. *Hum Brain Mapp* 2018;**39**(2).
